# Luminogens for Aggregation‐Induced Emission via Titanium‐Mediated Double Nucleophilic Addition to 2,5‐Dialkynylpyridines: Formation and Transformation of the Emitting Aggregates

**DOI:** 10.1002/chem.201905611

**Published:** 2020-02-21

**Authors:** Francesco Foschi, Kevin Synnatschke, Sebastian Grieger, Wen‐Shan Zhang, Hubert Wadepohl, Rasmus R. Schröder, Claudia Backes, Lutz H. Gade

**Affiliations:** ^1^ Institute of Inorganic Chemistry Heidelberg University Im Neuenheimer Feld 270 69120 Heidelberg Germany; ^2^ Applied Physical Chemistry Heidelberg University Im Neuenheimer Feld 253 69120 Heidelberg Germany; ^3^ Centre for Advanced Materials Heidelberg University Im Neuenheimer Feld 225 69120 Heidelberg Germany

**Keywords:** aggregation-induced emission, extinction spectroscopy, one-pot tetraarylation, scattering exponent, titanium tetraisopropoxide

## Abstract

New luminogens for aggregation‐induced emission (AIE), which are characterized by a branched cross‐conjugated 2,6‐bis(1,2,2‐triarylvinyl)pyridine motif, have been synthesized exploiting the one‐pot Ti‐mediated tetraarylation of 2,6‐bis(arylethynyl)pyridines. Thin layer solid‐state emitters were prepared by spin‐coating of the luminogens, while AIE‐colloidal dispersions were investigated in terms of optical density and scattering behaviour. This has given insight into particle size distributions, time evolution of the aggregation and the influence of different functionalization patterns on the luminescence of molecular aggregates. In particular, a combination of extinction spectroscopy and dynamic light scattering is being proposed as a powerful method for investigating the dynamic aggregation process in AIE‐type colloids.

## Graphical abstract

**Aggregate and shine**! New luminogens for aggregation‐induced emission (AIE) have been synthesized exploiting the one‐pot Ti‐mediated tetraarylation of 2,6‐bis(arylethynyl)pyridines. Their aggregation‐related photophysics and viscochromicity have been studied in detail.

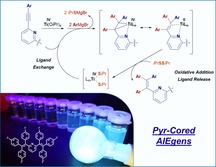

## Introduction

The development of highly efficient organic solid‐state emitters for both fluorescent and electroluminescent light sources is an important focus of contemporary materials science. While being potent emitters in dilute solutions, for many common polycyclic aromatic fluorophores the emission is at least partially quenched at higher concentration. The formation of aggregates, which may inter alia involve π–π stacking of aromatic rings, generally results in aggregation‐caused quenching (ACQ) of fluorescence and many efforts, including functionalization of the fluorophores with bulky residues, have been described over the years to overcome this limitation.^1^, ^2^, ^3^, ^4^ In addition, a different class of aromatic emitters, that luminesce upon aggregation, was first reported by Tang and collaborators about twenty years ago, including in particular propeller‐shaped tetraphenylethylene (TPE)^4^, ^5^, ^6^, ^7^ and hexaphenylsilole (HPS)^8^, ^9^, ^10^ derivatives, which are non‐emissive in common polar solvents but display intense fluorescence when an “antisolvent” is added which induces molecular aggregation. Intramolecular rotation and vibration, which enables non‐radiative relaxation pathways in solution, are thought to be restricted upon aggregation, thus leading to AIE.^11^, ^12^, ^13^, ^14^ Solid‐state and colloidal luminogens, including HPS and TPE derivatives, have been exploited for various analytical applications, including detection of metal ions,^15^, ^16^, ^17^, ^18^, ^19^ pH, small molecules,^20^, ^21^, ^22^, ^23^, ^24^, ^25^, ^26^, ^27^ and biomolecules,^28^, ^29^, ^30^, ^31^, ^32^, ^33^, ^34^, ^35^, ^36^, ^37^, ^38^, ^39^, ^40^ as well as mechanofluorochromic sensing.^41^, ^42^

**Scheme 1:**
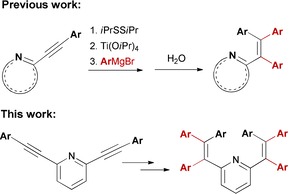
Ti(O*i*Pr)_4_‐mediated multiple aryl Grignard addition to alkynylated N‐heterocycles based on a 2‐alkynylpyridine motif, giving 2‐(1,2,2‐triarylvinyl)‐pyridines.

We recently developed a Ti(O*i*Pr)_4_‐mediated double aryl Grignard addition to 2‐alkynylpyridines and related alkynylated N‐heterocycles, which could be employed for a one‐pot synthesis of 2‐(1,2,2‐triarylvinyl)‐pyridines (Scheme 1).^43^

The method was a conceptual extension of earlier work on the carbo‐ and azaphilic Grignard addition to the nitrile group in *ortho*‐cyanido N‐heterocycles.^44^ The originally reported protocol could now be adapted to provide a straightforward synthetic access to 2,6‐bis(1,2,2‐triarylvinyl)‐pyridines in two steps from 2,6‐dibromopyridine via Sonogashira coupling and subsequent double aryl Grignard addition to the C≡C triple bonds.

The target compounds were found to be AIE‐type luminogens, to an extent which was found to depend on the aryl substituents. The aim of this work has been the investigation of the stability and optical properties of the emitting aggregates using a combination of fluorescence spectroscopy and optical extinction spectroscopy. Moreover, their time‐dependent evolution has been studied by analyzing the scattering contribution to the extinction spectra combined with dynamic light scattering (DLS). Finally, the AIE properties of the colloidal dispersions have been compared with the alternative, aggregation independent, viscochromic enhancement of the emission intensity.

## Results and Discussion

### Synthesis and structural characterization of the new 2‐(1,2,2‐triarylvinyl)‐pyridine‐based AIE luminogens

**Scheme 2:**
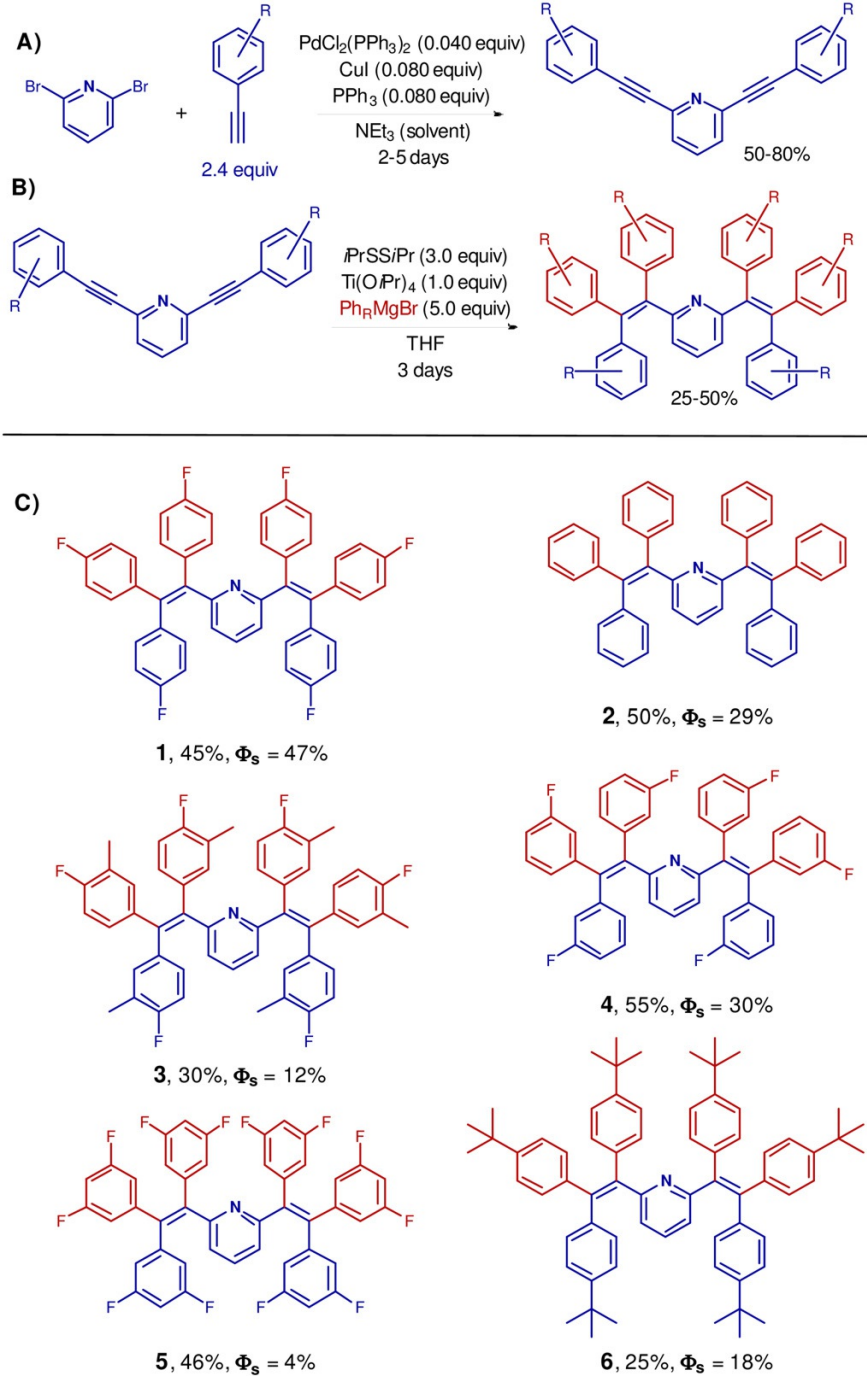
(A) Synthesis of V‐shaped 2,6‐bis(arylethynyl)pyridines by double Sonogashira coupling. (B) Synthesis of **1**–**6** by Ti(O*i*Pr)_4_‐mediated quadruple aryl Grignard addition to 2,6‐bis(arylethynyl)pyridines. (C) Compounds **1**–**6**; given are isolated yields and the emission quantum yields of spin‐coated films.

Based on our previous work on metal mediated nucleophilic additions to alkynes and nitriles, the Ti(O*i*Pr)_4_‐mediated tetraarylation of 2,6‐bis(arylethynyl)pyridines was applied to the synthesis of 2,6‐bis(1,2,2‐triarylvinyl)pyridines, as shown in Scheme 2.^43^

**Scheme 3:**
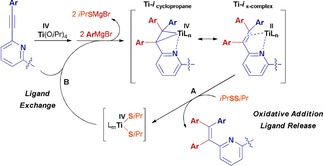
Proposed role of isopropyl disulfide in the titanium‐mediated tetraarylation of V‐shaped 2,6‐bis(arylethynyl)pyridines. (A) Liberation of the product and regeneration of a titanium(IV) species by oxidative addition of isopropyl disulfide. (B) Regeneration of a titanacyclopropane by ligand exchange and double aryl Grignard addition to the 2‐alkynylpyridine motif.

From the commercially available 2,6‐dibromopyridine a straightforward 2‐step protocol was employed for the synthesis of target compounds **1**–**6**: a double Sonogashira coupling with differently substituted arylacetylenes followed by Ti(O*i*Pr)_4_‐mediated quadruple nucleophilic arylation of the bis(alkynes). This yielded the 2,6‐bis(1,2,2‐triarylvinyl)pyridine derivatives **1**–**6** (Scheme 2 C). In particular, the use of a mild and soft oxidizing agent, such as isopropyl disulfide, within a one‐pot reaction protocol allows for the use of the Ti reagent either stoichiometrically or substoichiometrically, the latter giving rise to the optimized reaction protocol.^43^ The titanium‐mediated double arylation of each 2‐(arylethynyl)pyridine branch results in the generation of titanacyclopropanes (**Ti‐i_cyclopropane_**) with a strong titanium(II) π‐complex character (**Ti‐i_π‐complex_**) (Scheme 3).^43^ The organic ligand, which is the product of double aryl Grignard addition, can be liberated by oxidative addition of isopropyl disulfide (Scheme 3 A), thus allowing for the regeneration of a titanium(IV) center that can undergo a new reactive cycle by ligand exchange (Scheme 3 B). This type of reactive behaviour is key to achieving the desired one‐pot tetraarylation of 2,6‐bis(arylethynyl)pyridines under mild conditions.

**Figure 1:**
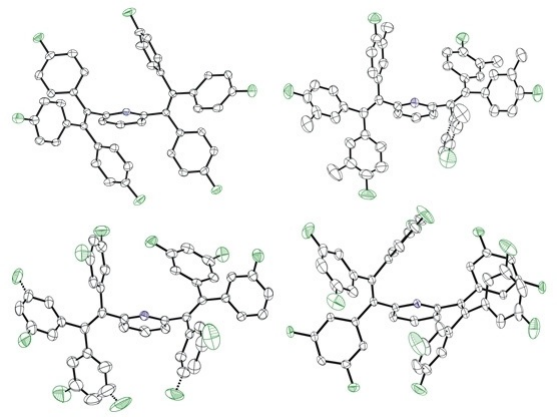
Molecular structures of the 4‐fluorophenyl substituted compound **1** (top, left), the 3‐methyl‐4‐fluorophenyl substituted compound **3** (top, right), the 3‐fluorophenyl substituted compound **4** (bottom, left) and the 3,5‐difluorophenyl substituted compound **5** (bottom, right), illustrating the propeller‐shaped geometries of the tetraarylethylene units involved [fluorine atoms: green; nitrogen atoms: blue].

The detailed geometries of the propeller‐shaped tetraarylethylene units in **1**–**5** were determined by single crystal X‐ray diffraction (Figure 1 and Supporting Information). Disorder in the crystal structures of **3** and **4** reflects alternative orientations of 3‐methyl‐4‐fluorophenyl and 3‐fluorophenyl groups, respectively.

The propeller‐shaped arrangements of the tetraarylethylene units observed in the crystal structures of all five compounds are consistent with the previously identified structural criteria for the observation of AIE. The presence of the *para*‐fluorinated phenyl groups on the pyridine‐cored backbone of **1** is associated with the highest luminescence quantum yield (Φ_S_=47 %, Scheme 1) in the **1**–**6** series. In the structure of **1**, ethylene C=C bond lengths of 1.348–1.354 Å and C−F bond lengths of 1.360–1.365 Å fall within the range previously reported for aromatic luminogens of related structure (Table 1).^45^

**Table 1:** Selected inter‐ and intramolecular interactions in single crystals of **1**.

X⋅⋅⋅Y	Distance (H⋅⋅⋅Y) [Å]	Distance (C⋅⋅⋅Y) [Å]	Angle (C−H⋅⋅⋅Y) [°]
C(18)−H(18)⋅⋅⋅F(6)_inter_	2.443	3.281	146.93
C(44)−H(44)⋅⋅⋅F(2)_inter_	2.634	3.544	160.76
C(45)−H(45)⋅⋅⋅F(4)_inter_	2.584	3.389	142.64
C(3)−H(3)⋅⋅⋅F(5)_inter_	2.669	3.305	124.85
C(4)−H(4)⋅⋅⋅F(1)_inter_	2.799	3.395	121.67
C(42)−H(42)⋅⋅⋅F(1)_inter_	2.785	3.406	123.75
C(33)−H(33)⋅⋅⋅Cg(1)_intra_	3.043	3.982	169.98
C(13)−H(13)⋅⋅⋅Cg(2)_intra_	3.248	4.164	162.58

As recently noted for fluorinated aromatic AIE‐luminogens, intermolecular aggregation based on weak F⋅⋅⋅H and C−H⋅⋅⋅π interactions may give rise to ordered supramolecular units, which in turn restricts the roto‐vibrational freedom of the aryl groups.^45^ This effect may counterbalance the higher tendency of fluorinated aromatics to undergo π–π stacking‐mediated ACQ, resulting in an overall enhanced fluorescence intensity upon aggregation.^4^, ^45^, ^46^

**Figure 2:**
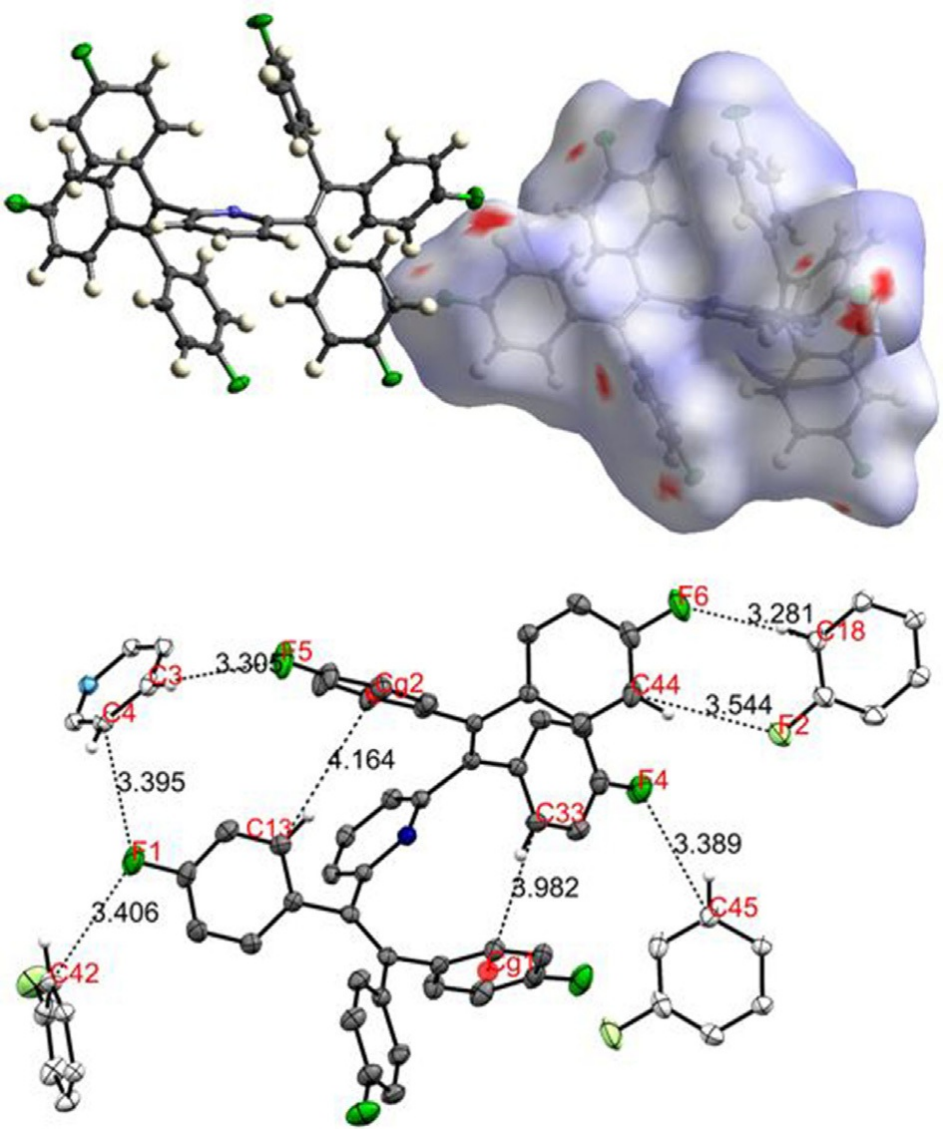
Hirschfeld surface mapped over d_norm_ (top), showing the length of intermolecular contacts as a color gradient between red (shortest contacts) and blue (longest contact). Propeller‐shaped structure of **1** (bottom), where selected intra‐ and intermolecular contacts have been highlighted for clarity. Cg1: Centroid (C20, C21, C22, C23, C24, C25); Cg2: Centroid (C34, C35, C36, C37, C38, C39).

Two intramolecular C−H⋅⋅⋅π contacts (C(33)⋅⋅⋅Cg1 and C(13)⋅⋅⋅Cg2 distances of 3.982 and 4.164 Å, respectively, Table 1) and several intermolecular C−H⋅⋅⋅F interactions of similar strength may be viewed as a particularly effective source for restriction of intramolecular motion (RIM) and consequent boost of the luminescence quantum yield (Figure 2).

In contrast, in spite of a loose packing of the aromatic rings in the solid state, the observed intra‐ and intermolecular contact distances are still consistent with moderate ACQ due to π–π interactions.^47^ The overall fluorescence intensity (vide infra) can therefore be seen as the net outcome of competing RIM and ACQ processes upon aggregation.^45^ In order to visualize the intermolecular interactions in the crystal, normalized contact distances (d_norm_) were mapped onto the molecular Hirschfeld surface (Figure 2).^48^, ^49^, ^50^, ^51^ As expected, the local minima of d_norm_ are often associated with fluorine substituents involved in weak intermolecular H⋅⋅⋅F bonds (Figure 2). The lack of fluorine substituents in **2** and therefore the absence of intermolecular H⋅⋅⋅F contacts is expected to result in a less pronounced RIM. Moreover, one of the six phenyl substituents of **2** is involved in parallel‐displaced π–π stacking with another phenyl group from a neighboring molecule (twist angle: 79.30°; Cg–Cg’ contact length: 3.868 Å, see Supporting Information, Figure S2). These findings may play a role in the reduced luminescence quantum yield of **2** (Φ_S_=29 %) compared to the performance of **1** (vide infra).

Finally, intermolecular interactions in the crystal network of the *meta*‐substituted **3**–**5** may be mostly related to intermolecular H⋅⋅⋅F contacts, with only minor contribution of C−H⋅⋅⋅π contacts. The observed luminescence quantum yields as low as Φ_S_ **(5)**=4 % and Φ_S_ **(3)**=12 % can also be interpreted as the consequence of reduced RIM due to a more pronounced disorder in the solid state and, at the same time, a possibly enhanced ACQ due to multiple functionalization of the aromatic rings.^52^

### The AIE properties of the *para*‐fluorophenyl substituted compound 1: Luminescence in a solvent/antisolvent system

**Figure 3:**
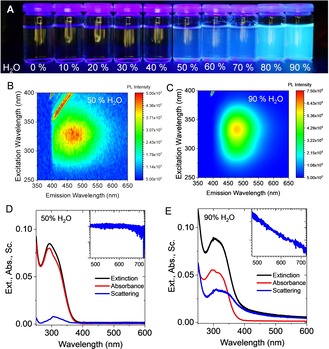
A) Photograph of solutions of **1** in different water/acetonitrile mixtures under UV irradiation showing a pronounced increase in photoluminescence intensity at water volume percentages above 50 %. B,C) Photoluminescence excitation contour plots of **1** in 50 % v/v H_2_O (B) and 90 % v/v H_2_O (C). d–E) Absorbance, extinction and scattering spectra of **1** in 50 % v/v H_2_O (D) and 90 % v/v H_2_O (E). The insets show the scattering in the non‐resonant regime on a double logarithmic scale. The linear decrease in (E) is characteristic of scattering.

To characterize the AIE‐type luminescence in more detail, we investigated aggregated colloids in liquid suspension. Aggregation was induced by adding water as an antisolvent to a solution of **1** in acetonitrile (Figure 3 A). The photoluminescence (PL) intensity of **1** was found to be negligible in diluted acetonitrile solutions, as well as in water/acetonitrile mixtures with a water content below 50 %. A strong monotonic increase of the PL intensity (up to a factor of ca. 300) was observed with increasing water content in mixtures ranging from 50 to 90 %, indicating aggregation‐induced emission. The extent of the boost in emission intensity can be extracted from photoluminescence excitation (PLE) contour plots of the mixtures with a water content of 50 and 90 % shown in Figure 3 B,C, respectively (for more data, see Supporting Information Figure S3). The emission maximum is centered at 470 nm in both cases, giving rise to the blue color in Figure 3 A. The maximum in the excitation shifts from ca. 310 nm for 50 % volume percent (v/v) of water to ca. 325 nm for 90 % H_2_O.

### Particle formation and AIE: Wavelength‐dependent optical extinction

Wavelength‐dependent optical extinction spectra can contain rich information on the number and size of particles due to light scattering in the non‐resonant regime. This approach was taken to analyze the evolution of the particle sizes in the aggregation of **1** and its impact on the PL response. The overall extinction *ϵ*(*λ*) of colloidal suspensions at a given wavelength *λ* arises as the sum of the absorption α(*λ*) and scattering σ(*λ*) [Eq. 1]:

ϵ(λ)=α(λ)+σ(λ)

Both components were deconvoluted with the aid of an integrating sphere which allowed to collect scattered light and thus to record the true absorbance spectra (for the latter, see also Supporting Information). The scattering background σ(*λ*) was determined as the difference between the measured extinction *ϵ*(*λ*) and absorbance values α(*λ*) at a given wavelength. Examples for 50 and 90 % v/v H_2_O mixtures are shown in Figure 3 D, E. The absorbance spectra of **1** are generally characterized by a broad band over a spectral range of 300–350 nm with an unresolved vibronic fine structure.

For the 50 % v/v H_2_O sample, absorbance and extinction measurements agree well (Figure 3 D) which is consistent with the absence of solid bodies in suspension and hence negligible scattering in this homogeneous system.

This contrasts with the spectra of the 90 % v/v H_2_O sample (Figure 3 E) in which about 1/3 of the measured extinction in the resonant regime is due to contributions from scattering. The scattering spectrum in the resonant regime is red‐shifted with respect to the pure absorbance, but similar in shape, leading to an apparent bathochromic shift of the peak positions.^53^, ^54^

In addition, scattering is manifested in the extinction spectra in the non‐resonant regime, where it decreases with wavelength in intensity as a power law.^53^, ^54^ The power law scaling can be best discerned when plotting the spectral region of the non‐resonant regime on a double logarithmic scale (insets in Figure 3 D–E), the linear scaling being characteristic for non‐resonant scattering. The aggregation process itself may be followed in more detail by recording time‐dependent extinction spectra.

### Time dependence of aggregation and particle formation

**Figure 4:**
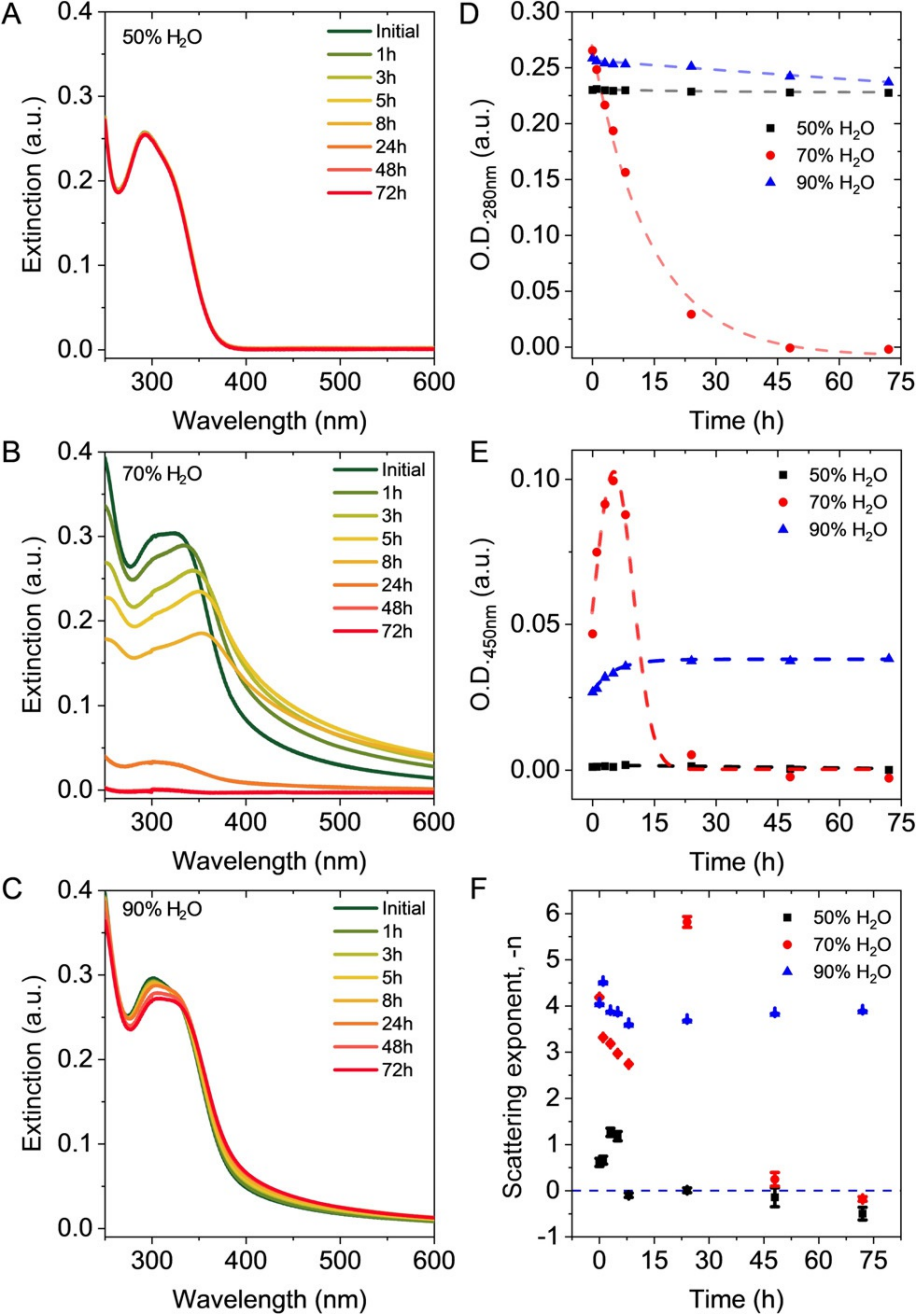
A–C) Optical extinction spectra of **1** in different acetonitrile/water mixtures measured at various time intervals after preparation. A) 50 % v/v H_2_O, B) 70 % v/v H_2_O, C) 90 % v/v H_2_O. D) Plot of the optical extinction at 280 nm (resonant regime) as function of time. E) Plot of the optical extinction at 450 nm (non‐resonant regime) as function of time. F) Plot of the scattering exponent (from 400–650 nm) as function of time. The horizontal broken lines in (D–F) are guides for the eye.

In Figures 4 A–C extinction spectra of **1** are shown, measured at various time intervals after preparing the acetonitrile/water mixtures (also see Supporting Information Figure S4).

Neither the extinction nor the barely detectable PL of **1** change over time in the water/acetonitrile=50:50 mixture (Figure 4 A) which is consistent with negligible aggregation. This solvent mixture may be considered as the threshold mixture triggering aggregation (see also Figure 3 A) in the water/acetonitrile system. In contrast, a strong apparent red‐shift of the main extinction signal from 330 nm to around 360 nm, a general broadening of the spectral profile and non‐negligible extinction in the non‐resonant regime (*λ*>400 nm, due to scattering) take place within a few hours after the preparation of the sample for 70 % v/v H_2_O (Figure 4 B). Finally, a moderate red‐shift is observed over time in the 90 % v/v H_2_O mixture, together with a slight increase of the optical extinction in the non‐resonant regime at high wavelengths (Figure 4 C).

The extinction in the resonant regime can be used to monitor the amount of **1** forming sediment in the suspension because the scattering follows the absorbance in shape, and therefore extinction (as well as absorbance) can be used to track the concentration of **1**. Figure 4 D depicts the time‐dependence of the extinction at 280 nm, that is, the resonant regime, indicating that concentration of dissolved **1** drops roughly exponentially with time in the 70 % v/v H_2_O solvent mixture, while it is essentially invariant for both the 50 % v/v H_2_O and 90 % v/v H_2_O solvent mixtures.

In addition, the measurement of optical extinction at a given wavelength in the non‐resonant regime can be used to indicate the evolution of the scattering strength.^53^ Figure 4 E displays a plot of the extinction at 450 nm as a function of time. In the 50 % v/v H_2_O solvent mixture, the extinction remains approximately zero due to the absence of larger aggregates. In contrast, the extinction in the 70 % v/v H_2_O solvent mixture increases at first, before falling off rapidly at *t*>8 h. This suggests the initial formation of large aggregates (with higher scattering strength) in the first few hours, which then precipitate. This gives rise to the drop in extinction as well as the decreasing chromophore concentration observed in Figure 4 D in the resonant regime for this solvent mixture. In contrast, the extinction at 450 nm increases only slightly over time in the 90 % v/v H_2_O mixture, suggesting a slight increase in aggregate content (or size) with time, mirroring the essentially constant extinction in the resonant regime (Figure 4 D). Both observations indicated the presence of an overall stable colloid in that particular solvent mixture.

It is possible to derive information on aggregate size by analyzing the scattering exponent in the non‐resonant regime which changes systematically with particle size, a smaller exponent indicating the formation of larger particles and vice versa.^53^ To this end, the extinction in the 400–650 nm range was plotted on a double logarithmic scale and fitted by linear regression (Supporting Information Figure S5). The slope of this fit corresponds to the respective negative scattering exponent, and the time evolution of these exponents for the different solvent mixtures (50, 70 and 90 % v/v H_2_O in acetonitrile) are shown in Figure 4 F.

Scattering exponents are in the range of 2 and 4 as would be expected from Mie theory^53^ (see Supporting Information for details). In the case of the 70 % v/v H_2_O solvent mixture, a sharp decrease in the scattering exponent is observed within the first 8 h after mixing the solvents, implying a rapid growth of particles, which eventually precipitate from the suspension. This accounts for the decrease in extinction in both the resonant (Figure 4 D) and non‐resonant (Figure 4 E) regime noted above. In case of the 90 % v/v H_2_O solvent mixture, the scattering exponent drops slightly before equilibrating at 8 h which is consistent with a slow and minor growth of the suspended particles. Overall, these findings suggest the formation of larger particles in solvent mixtures with an intermediate water content of 70 %, which is consistent with previous reports by Tang and collaborators for related aromatic AIE‐luminogens.^8^, ^55^

### Determination of particle size distributions by dynamic light scattering

**Figure 5:**
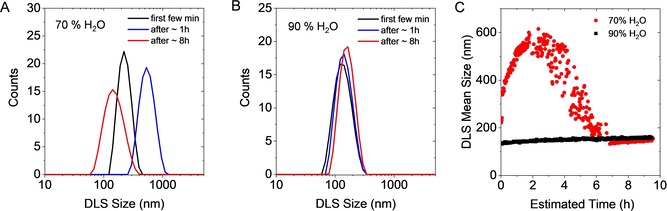
Aggregate size according to dynamic light scattering as function of time of **1** in two different acetonitrile/water mixtures. A) DLS size for the 70 % v/v H_2_O solvent mixture. B) DLS size for the 90 % v/v H_2_O solvent mixture. C) Evolution of the DLS size as function of time for both mixtures.

To obtain additional evidence for the conclusions proposed above, dynamic light scattering (DLS) measurements were conducted on **1** in 50 %, 70 % and 90 % v/v H_2_O solvent mixtures as a function of time. A subset of the data is shown in Figure 5 (the complete data set is provided in the Supporting Information Figure S6). In good agreement with the analysis of the extinction spectra, DLS confirms that the mean particle size of the colloids of **1** increases within the first two hours from 215 nm to 530 nm in the case of the 70 % v/v H_2_O solvent mixture (Figure 5 A). After 8 h, the average size of the colloids is smaller (145 nm) than the initial size. The extinction spectra suggest that this is due to selective precipitation of larger aggregates, leaving a minor fraction of smaller particles in suspension. In contrast, a moderate increase of the mean size of the colloids of **1** is observed for the 90 % v/v H_2_O solvent mixture (Figure 5 B) from ≈130 nm to ≈160 nm. Importantly, even such a subtle change in aggregate size can be well captured by changes in the scattering coefficient as represented in Figure 4 E, F. The overall evolution of particles size over time is shown in Figure 5 C.

Overall, DLS strongly supports the conclusions drawn from the extinction spectroscopic analysis, demonstrating the rich information encoded in optical extinction spectroscopy and its potential to systematically investigate aggregate formation of discrete molecules.

### Optical and scanning electron microscopy of the particles formed in the aggregation process

**Figure 6:**
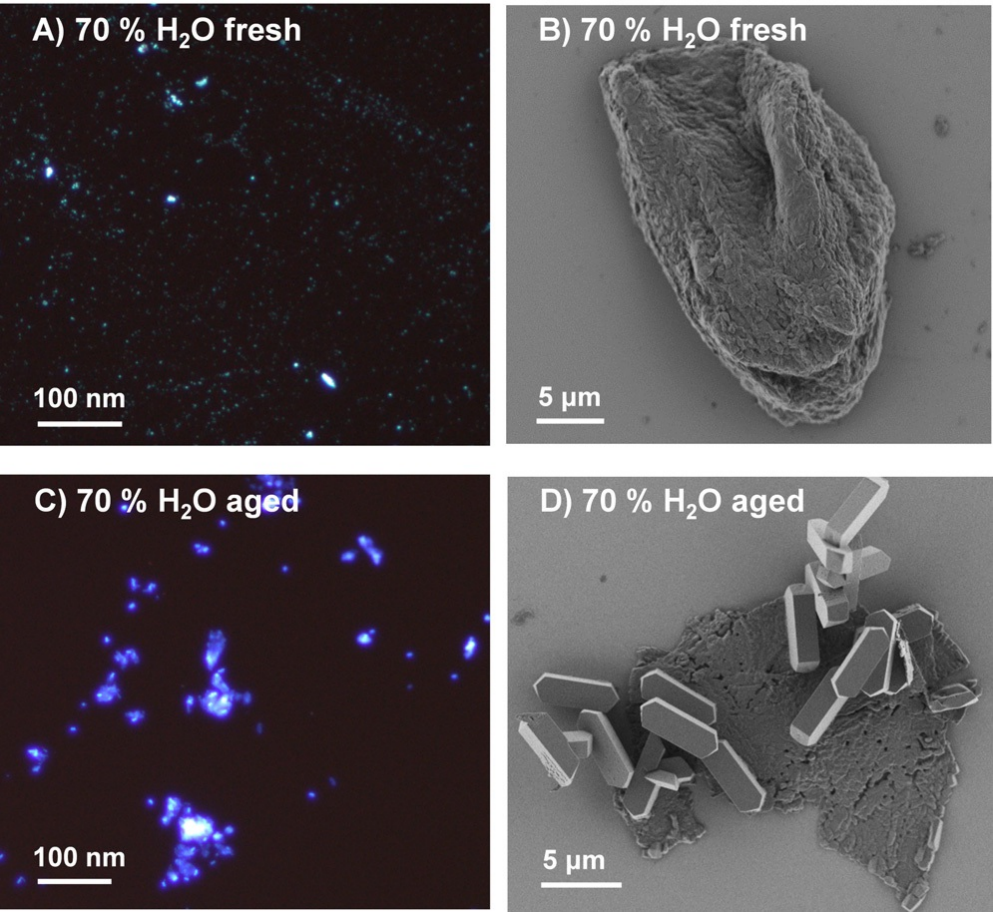
Solid amorphous particles of **1**. A–B) Freshly prepared water:acetonitrile=70:30 suspension imaged with optical microscopy (A, excitation wavelength: 385 nm) and SEM image (B). C–D) Images after 48 h of incubation at room temperature. The presence of larger aggregates emitting at lower wavelengths is observed in optical microscopy (C, excitation wavelength: 385 nm), while crystalline and thin layered particles of **1** are seen in SEM (D).

Optical microscopy (OM) and scanning electron microscopy (SEM) further confirmed the findings discussed above. In a freshly prepared mixture of **1** in a 70 % v/v H_2_O mixture, optical microscopy confirmed the presence of small, uniformly distributed, luminescent deposits along with few bigger particles (Figure 6 A). According to SEM, these initial aggregates are amorphous (Figure 6 B). During incubation of the suspension at room temperature, the average size of the aggregates increased and the fluorescence emission blue‐shifted, according to optical microscopy (Figure 6 C). Micrometer sized solid particles of **1**, including crystals of regular shape and dimensions, were collected together with essentially bi‐dimensional aggregates (Figure 6 D) from the mixture with a 70 % v/v H_2_O by drop casting under mild conditions. Notably, the fraction of crystalline material in the sample was found to grow over time and accompanied the observed separation and sedimentation of solid material from the mixture.

**Figure 7:**
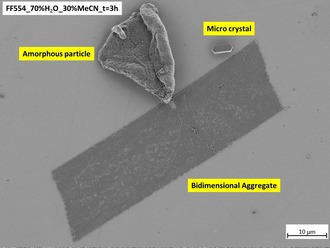
Amorphous, crystalline and bi‐dimensional aggregates after three hours of incubation in a 70 % v/v H_2_O mixture at room temperature.

Amorphous, crystalline and bi‐dimensional aggregates coexist in the system three hours after preparation of the mixture, as clearly shown in Figure 7.

In summary, the characterization of the colloidal systems formed from **1** in acetonitrile/water mixtures established the aggregation‐induced emission, with aggregate size scaling non‐linearly with the content of the antisolvent and a characteristic time evolution. In each case, the PL data can be heavily influenced by changes in concentration and particle size.

### Aggregation‐induced emission versus viscochromicity

As discussed above, the observed AIE of the 2,6‐bis(1,2,2‐triarylvinyl)pyridines may be understood in terms of frozen intramolecular motion upon aggregation and thus suppression of associated non‐radiative deactivation pathways. An alternative approach to slow down intramolecular mobility is based on the increase in viscosity of the surrounding medium, that is, the solvent system. We therefore compared the response of solutions of compound **1** to anti‐solvent addition, on the one hand, and to addition of a viscous co‐solvent, on the other.

**Figure 8:**
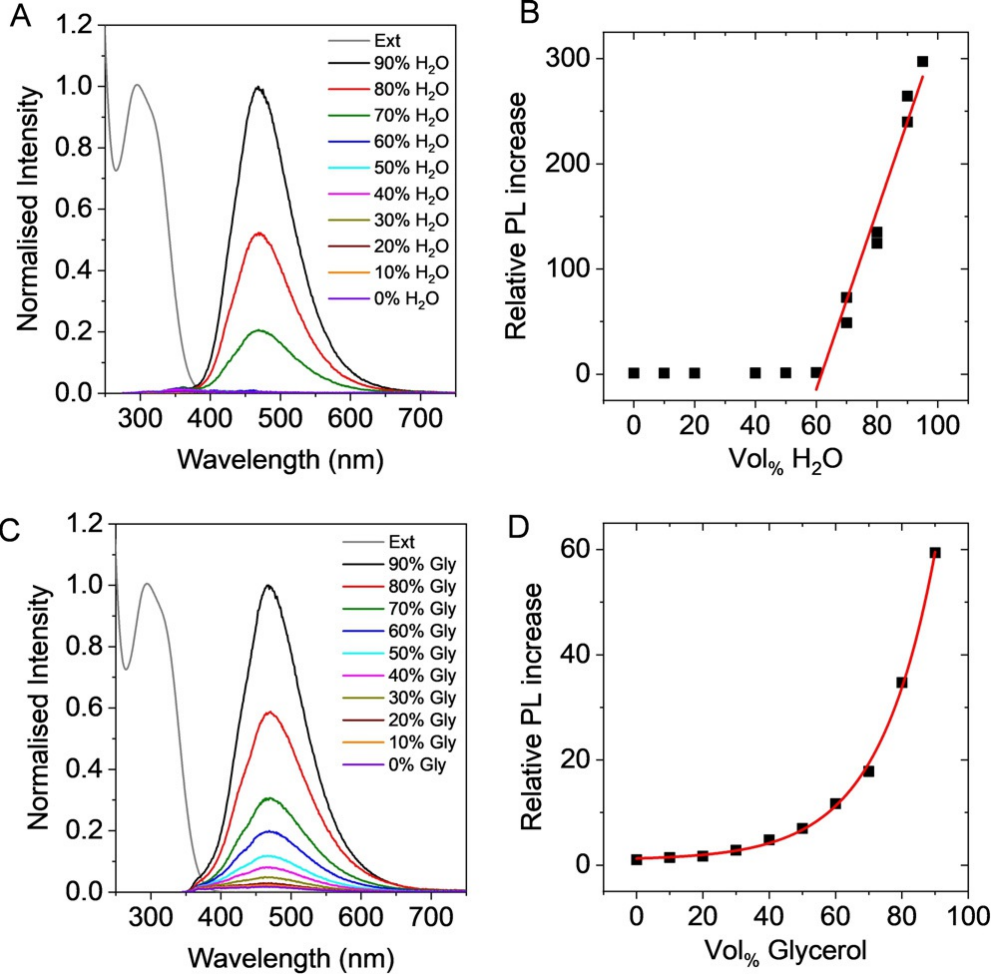
A–B) Aggregation induced emission of **1** enforced by the addition of water to **1** dissolved in acetonitrile. A) PL spectra (excitation at 330 nm) normalized to the maximum at 90 % v/v H_2_O. The normalized absorbance spectrum of **1** in acetonitrile is added. B) Evolution of the PL intensity as function of water volume fraction normalized to the PL intensity in acetonitrile. C–D) Viscochromic luminescence enhancement of **1** enforced by addition of glycerol to increase solvent viscosity. C) Normalized PL spectra (excitation at 330 nm). D) Evolution of the PL intensity as function of glycerol volume fraction normalized to the PL intensity in DMSO.

Figure 8 A, B summarizes the aggregation‐induced emission of **1** in water/acetonitrile mixtures immediately after preparation of the samples. As seen visually from the photograph in Figure 3 A, the PL increases abruptly after a certain threshold of water content, when aggregation occurs (Figure 8 A). Above a threshold of 60 %, a linear increase in PL intensity is observed (Figure 8 B). The investigations described above clearly demonstrated that aggregates are larger at intermediate water contents, on the one hand, and that a linear scaling of PL with increasing volume fraction of water can be observed, on the other. This strongly suggests that the PL enhancement is independent of the size of aggregates and rather a fingerprint of aggregation strength, that is, intermolecular interaction, which can be understood in terms of RIM.

As indicated, such a RIM of AIE‐type luminogens may also be induced by increasing the viscosity of the solvent system. In this case, aggregation‐independent fluorescence enhancement arises from the restriction of rotational and vibrational intramolecular motion, resulting from increased solvent viscosity (Figure 8 C, D).^8^, ^55^

This aggregation‐independent viscochromic behaviour of **1** was investigated measuring the PL intensity of its DMSO solution upon addition of different amounts of glycerol, the viscosity of the latter being approximately 700 times greater than that of the former. The fluorescence intensity of the solution indeed increased upon increasing the amount of glycerol in the mixture (Figure 8 C and Supporting Information Figure S7). In contrast to AIE, which occurs abruptly at a given threshold in the antisolvent/solvent ratio (Figure 8 B), the PL increase displayed exponential dependence on the glycerol content in the range 20–90 %, as indicated by the exponential fit in Figure 8 D.

The classic Grunberg–Nissan mixing rule predicts an exponential relationship between the relative amount of solvents in the ideal mixture and the viscosity of the system.^56^ Thus, the changes in PL intensity can be easily and quantitatively linked to changes in viscosity in the nearly ideal DMSO/glycerol mixture, with the potential of viscometric application of the luminogen.^57^ In absolute terms, the photoluminescence is enhanced by a factor of >300 through the formation of aggregates with aid of water as antisolvent, while the viscochromic effect is significantly weaker resulting in a 60‐fold increase compared to the initial solution.

### Influence of *para*‐ and *meta*‐substitution patterns on the aggregation

Having analyzed the aggregation‐induced emission of compound **1**, we performed a comparative study for the 2,6‐bis(1,2,2‐triarylvinyl)pyridine derivatives **1**–**5** assessing the formation of the colloidal aggregates of all five compounds in terms of the magnitude of the PL increase and the water content threshold for aggregation (Supporting Information Figure S8–14).

**Figure 9:**
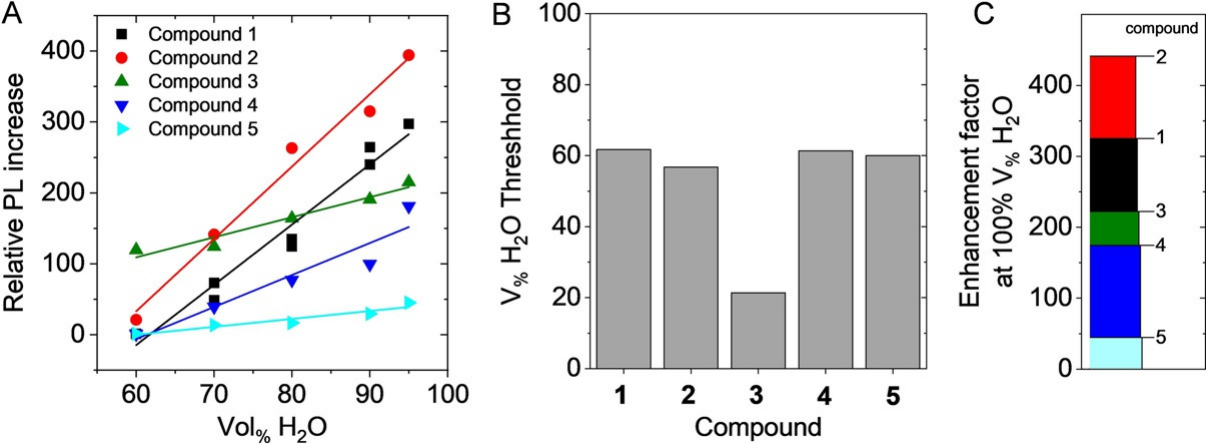
A) Evolution of the PL intensity as a function of water volume fraction, normalized to the PL intensity, in acetonitrile for compounds **1**–**5**. B) PL threshold of AIE. (C) Enhancement factor extrapolated from the fits in (A) for the different compounds at 100 % water content.

Figure 9 A displays a plot of the relative PL increase of all compounds under study as a function of water content, determined immediately after addition of the anti‐solvent. As delineated above, the data could be fitted to a linear function above the aggregation threshold, allowing the determination of the onset at which aggregation occurred and the enhancement factor (extrapolated to 100 % water content, Figure 9 C). The onset of AIE was found to occur at around 60 % v/v H_2_O (Figure 9 B) in all cases, except compound **3**, thus suggesting reduced solubility of the 4‐fluoro‐3‐methylphenyl functionalized **3** even at very low water content. However, the magnitude of PL enhancement (Figure 9 C) was found to be strongly dependent on the structure, the largest PL enhancement factor of >400 being found for the phenyl‐based luminogen **2**, followed by the *para*‐fluoro substituted **1** with an enhancement >300. *Meta*‐functionalization of the aryl groups in **3**, **4** and **5** appeared to hamper the increase of the PL intensity upon aggregation to some extent.

Notably, coexistence of two conformers of **4** in a C_6_D_6_ solution was shown by ^19^F{^1^H} NMR (see Supporting Information Figure S1). Moreover, in the X‐ray diffraction structures of **3** and **4**, disorder in the *meta*‐functionalized aryl groups indicated the coexistence of different rotamers which differ by a 180° rotation of the rings about their spinning axis (Figure 1). Such disorder can obviously not result from rotation of the aryl groups in **1** and **2** for symmetry reasons.

*Meta*‐functionalization of the aryl rings appears therefore to be associated with a less pronounced increase of fluorescence intensity upon aggregation and lower luminescence quantum yields for spin‐coated solid films, as expected in case of reduced RIM in the solid state. Notably, the *meta*‐arrangement of the fluoro‐substituents on aryl substituents of **4** and **5** did not have a strong impact in the evolution of the extinction spectra of the dispersion compared to the *para*‐fluorinated aryl substituents of **1**, while major deviations were observed for **2** and **3**.

**Figure 10:**
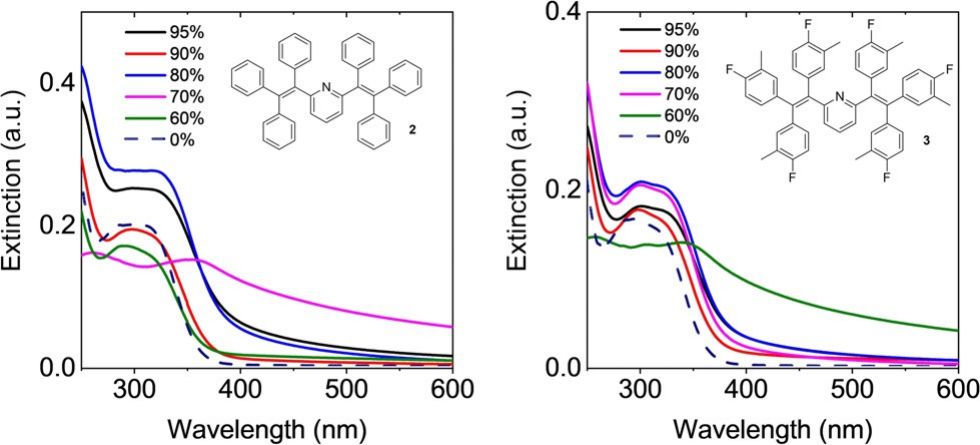
Time‐zero extinction spectra of **2** (left) and **3** (right) in acetonitrile/water mixtures.

In particular, a water content of 70 or 60 % in the solvent mixture induced a dramatic change in the time‐zero spectral profile of **2** and **3**, respectively, when compared to the corresponding spectra of **1**, which consisted of an apparent red‐shift of the main extinction signal from 330 to 345–355 nm, a general broadening of all the signals and very strong extinction in the non‐resonant regime (Figure 10). These features are a clear fingerprint of a significant contribution of light scattering to the extinction spectra. Generally, the presence of phenyl (**2**) or 4‐fluoro‐3‐methylphenyl (**3**) substituents appears to result in a more rapid formation of highly scattering suspensions, in comparison to **1**.

**Figure 11:**
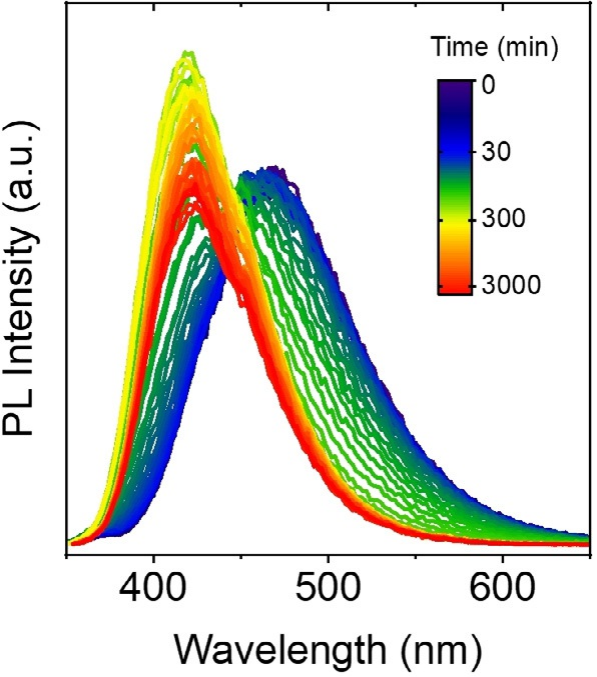
Time evolution of PL emission of **3** in a water/acetonitrile mixture with a 70 % v/v H_2_O.

In addition, a pronounced blue‐shift in the fluorescence emission spectra of **2**, **3** and **4** was observed over time in mixtures with an intermediate water content (60–80 %, Supporting Information Figures S11–13). The effect is particularly pronounced for **3**, where a blue shift of around 60 nm takes place gradually within the first 5 hours after preparation of the sample (Figure 11). Only limited sedimentation of fluorescent particles and no severe drop in the PL intensity were observed 48 h after preparation of the sample, as opposed to the corresponding suspension of **1**, thus suggesting the formation of a relatively stable colloid of **3** at intermediate water contents. This is consistent with the main features of the optical extinction profile of the system, where a gradual shift of the main extinction band towards longer wavelengths and an increase of the scattering background in the non‐resonant regime can be observed in aged samples (24 h, 48 h) of **3** as a result of the absence of substantial sedimentation of the luminogen (see Supporting Information Figure S12).

Finally, the comparison between the wavelength of maximum emission of **1**–**4** as colloidal dispersions and spin‐coated thin films is presented in Table 2. With the exception of **2**, the spin‐coated and colloidal luminogens with a 90 % water content are characterized by emission maxima at comparable wavelengths, thus suggesting similar molecular and packing geometries in the two aggregated states, both resulting from rapid aggregation of the luminogen on the one hand. On the other hand, in line with what was observed for **1** and **3** (Figure 11), emission of colloids of **4** with a 70 % water content and of **2** with a 80 % water content (see Supporting Information Figures S11, S13) is blue‐shifted, as expected for a different, possibly crystalline character of slowly forming aggregates.^4^

**Table 2:** Selected emission maxima of **1**–**4**.

	*λ*_MAX_(1) [nm]	*λ*_MAX_(2) [nm]	*λ*_MAX_(3) [nm]	*λ*_MAX_(4) [nm]
Film_SPIN‐COATED_	464	493	468	464
90 % v/v H_2_O _t0_	471	479	471	468
90 % v/v H_2_O _24 h_	466	474	468	470
70 % v/v H_2_O _t0_	466	471	466	452
70 % v/v H_2_O _24 h_	463	470	420	445

## Conclusions

The titanium‐mediated one‐pot quadruple aryl Grignard addition to 2,6‐bis(arylethynyl)pyridine triple bonds has been employed as a convenient method to synthesize various bis(1,2,2‐triarylvinyl)pyridine derivatives that act as luminogens for aggregation‐induced emission. The synthetic method itself is straightforward and may be extended to other target compounds in future studies.

The colloidal dispersions of the luminogens have been investigated and thereby insight into particle size distributions, time evolution of the aggregation and physical stability of the suspensions was obtained. Spin‐coating of the luminogens in solid films was used for the preparation of solid‐state emitters with low to moderate quantum yields. Finally, an exponential aggregation‐independent relationship between viscosity of the medium and fluorescence intensity has been determined.

It is evident that the exploration of the optical properties of AIE‐type colloids by straightforward spectroscopic methods may provide the basis for a better understanding and a finer tuning of AIE‐based analytical methods of practical relevance, thus improving reliability and reproducibility of AIE‐sensing systems. This will be the aim of future work on these systems in our laboratory.

## Experimental Section

All reactions were carried out in oven‐dried flasks (150 °C) under dry inert gas atmosphere, according to standard Schlenk techniques. ^1^H and ^13^C{^1^H} NMR spectra were recorded in [D_6_]benzene at room temperature on a Bruker Avance II (400 MHz) or a Bruker Avance III (600 MHz) spectrometers. Chemical shifts are reported in parts per million (ppm) and referenced internally to the benzene residual proton or carbon signals (*δ*=7.16 or *δ*=128.06 ppm, respectively). ^19^F{^1^H} NMR spectra were recorded in [D_6_]benzene at room temperature on a Bruker Avance II (400 MHz). Chemical shifts are reported in parts per million (ppm) and referenced to an external standard (CFCl_3_). High resolution mass spectra were acquired on Brucker ApexQe Hybrid 9.4 T FT‐ICR and JEOL JMS‐700 magnetic sector (EI, LIFDI) spectrometers.

**X‐ray diffaction study**: Full shells of intensity data of single crystal X‐ray diffraction analysis were collected at low temperature with a Bruker AXS Smart 1000 CCD diffractometer (Mo‐*K*_α_ radiation, sealed X‐ray tube, graphite monochromator) or an Agilent Technologies Supernova‐E CCD diffractometer (Mo‐*K*_α_ radiation, microfocus X‐ray tube, multilayer mirror optics). Molecular structures of **1, 3**–**5** have been included in the main text: Nitrogen atoms: blue. Fluorine atoms: green. Thermal ellipsoids set at the 50 % probability level. H‐atoms have been omitted for clarity. For the discussion of compound **1** (Table 1, Figure 2) bond lengths and angles were calculated with normalized hydrogen positions.^58^ For further details see Supporting Information.

**Starting materials**: Synthetic procedures of the double Sonogashira coupling for the synthesis of the precursors (2,6‐bis(arylethynyl)pyridines) have been included in Supporting Information. 2,6‐bis(phenylethynyl)pyridine and 2,6‐bis((4‐fluorophenyl)ethynyl)‐pyridine are known compounds and have been previously characterized.^43^

**Optical extinction and absorbance** measurements were carried out with an Agilent Cary 6000i spectrometer in quartz cuvettes. The spectrometer was equipped with an integrating sphere (external DRA‐1800) for absorbance measurements. In this case, the cuvettes were placed in the center of the sphere. The measurements of both, extinction and absorbance spectra allow for the calculation of scattering spectra (Sca=Ext−Abs). All spectra were acquired with 0.5 nm increments and 0.1 s integration time.

**Fluorescence measurements** were carried out in quartz cuvettes at 20 °C using a Fluorolog‐3 Horiba Scientific Fluorescence spectrometer equipped with a Syncerity PMT detector and a 450 W xenon light source for excitation. Typical acquisition parameters were 0.2 s integration time in a range of 350–645 nm with increments of 0.5 nm. Light at 330 nm was used for excitation and the selected bandpass was 3 nm for both, excitation and emission. The PLE maps were acquired with excitation wavelengths between 250 and 400 nm and emission between 350 to 650 nm. A 400 nm cut off filter was used to avoid second order harmonics for the PLE mapping.

**Solid state emission spectra** were recorded using an integrating sphere (diameter 6’’, coated with Spectraflect®) on a Jasco FP‐6500 spectrofluorometer. Spin‐coating of the luminogens from DCM solutions (10 mg mL^−1^; 25 °C, 1500 rpm) has been used for the production of thin films of **1**–**6** on regular glass. Photoluminescence Quantum Yields (PLQY, Φ_S_) were calculated with the FelixGX software (PTI). Experiments have been performed in the laboratory of Prof. Dr. Uwe H. F. Bunz, Institute of Organic Chemistry, Heidelberg University.

**Dynamic light scattering** experiments were conducted with a Zetasizer Nano ZSP from Malvern Panalytical equipped with a 633 nm HeNe laser. Measurements were performed in a glass cuvette at an 173° backscatter angle with general purpose analysis. The viscosity of water was assumed to be the sample viscosity due to the generally high fraction of water in the samples (1.0031 MPa s at 20 °C).

**Fluorescence micrographs** were recorded with a Zeiss Axio Imager 2 equipped with a Colibri LED light source for fluorescence illumination.

**SEM images** were obtained with a Zeiss Delta field‐emission scanning electron microscope using a landing energy of 500 eV and an Inlense detector. Probes were prepared on silicon wafers by drop‐casting in mild conditions, involving gentle and reiterated soaking of single droplets of the mixture, deposited on the same spot of the wafer surface, by use of absorbent filter paper at room temperature.

**General Procedure (GP‐1) for the preparation of 2,6‐bis(1,2,2‐triarylvinyl)pyridines**: To a solution of a 2,6‐bis(arylethynyl)pyridine in dry THF (2.0 mL for each mmol of pyridine) isopropyl disulfide (3.0 equiv) and Ti(O*i*Pr)_4_ (1.0 equiv) were added consecutively while stirring the solution at room temperature. After cooling the mixture to −78 °C, a THF solution of arylmagnesium bromide (5.0 equiv) was added dropwise, the cooling bath was then removed and the mixture was stirred at 40 °C for 3 days, hence turning black quickly. The reaction vessel was then cooled to room temperature in a water bath, distilled water (0.5 mL for each mmol of pyridine) was added dropwise and the mixture was stirred for 1 h. After addition of THF (5.0 mL) and dichloromethane (30 mL) the mixture was stirred vigorously for 2 h. The white precipitate was removed by filtration, the crude product was adsorbed over celite and purified by column chromatography (SiO_2_, eluent gradient: petroleum ether:ethylacetate=100:1 to 25:1, 0.25 vol% NEt_3_). After evaporation of the volatiles and recrystallization in ethyl acetate/pentane, products **1**–**6** were obtained as white solid. Recrystallization in benzene/pentane gave single crystals of **1**–**5** suitable for X‐ray diffraction analysis.

**Compound 1**: Colourless solid, 3.1 g, 4.5 mmol, 45 %. Alkyne: 2,6‐bis((4‐fluorophenyl)ethynyl)pyridine (3.2 g, 10 mmol); *i*PrSS*i*Pr (4.5 g, 4.8 mL, 3.0 equiv); Ti(O*i*Pr)_4_ (2.8 g, 3.0 mL, 1.0 equiv); ArMgBr: 4‐fluorophenylmagnesium bromide (1.0 m THF solution, 50 mL, 5.0 equiv). ^1^H NMR (600.13 MHz; C_6_D_6_; 295.2 K): *δ* [ppm]=6.86–6.55 (m, 21 H), 6.55–6.50 (m, 6 H). ^13^C{^1^H} NMR (150.90 MHz; C_6_D_6_; 295.7 K): *δ* [ppm]=162.3 (d, ^1^ *J*_CF_=247.4 Hz), 162.13 (d, ^1^ *J*_CF_=247.1 Hz), 162.05 (d, ^1^ *J*_CF_=247.4 Hz), 161.9, 140.5, 140.3, 139.4 (d, ^4^ *J*_CF_=3.3 Hz), 138.4 (d, ^4^ *J*_CF_=3.4 Hz), 138.0 (d, ^4^ *J*_CF_=3.4 Hz), 135.7, 133.12 (d, ^3^ *J*_CF_=7.8 Hz), 133.08 (d, ^3^ *J*_CF_=7.7 Hz), 132.7 (d, ^3^ *J*_CF_=7.8 Hz), 123.8, 115.3 (d, ^2^ *J*_CF_=21.5 Hz), 115.2 (d, ^2^ *J*_CF_=21.4 Hz), 114.9 (d, ^2^ *J*_CF_=21.2 Hz). ^19^F{^1^H} NMR (C_6_D_6_, 376.27 MHz, 295.0 K): *δ* [ppm]=−113.8, −114.18, −114.20. MS (HR‐DART(+)): calcd 696.2126 (C_45_H_28_F_6_N, [*M*+H]^+^), found 696.2116.

**Compound 2**: Colourless solid, 4.1 g, 7.0 mmol, Yield: 50 %. Alkyne: 2,6‐bis(phenylethynyl)pyridine (4.0 g, 14 mmol); *i*PrSS*i*Pr (6.3 g, 6.7 mL, 3.0 equiv); Ti(O*i*Pr)_4_ (4.0 g, 4.1 mL, 1.0 equiv); ArMgBr: phenylmagnesium bromide (1.0 m THF solution, 70 mL, 5.0 equiv). ^1^H NMR (399.89 MHz; C_6_D_6_; 295.3 K): *δ* [ppm]=7.14–6.77 (m, 30 H), 6.71–6.64 (m, 3 H). ^13^C{^1^H} NMR (100.55 MHz; C_6_D_6_; 295.2 K): *δ* [ppm]=162.4, 144.1, 143.3, 142.6, 142.4, 141.4, 135.3, 131.74, 131.67, 131.4, 128.1, 127.91, 127.88, 127.1, 126.79, 126.71, 123.9. MS (HR‐DART(+)):calcd 588.2691 (C_45_H_34_N, [*M*+H]+), found 588.2679.

**Compound 3**: Colourless solid, 0.42 g, 0.54 mmol, Yield: 30 %. Alkyne: 2,6‐bis((4‐fluorophenyl)ethynyl)pyridine (0.61 g, 1.8 mmol); *i*PrSS*i*Pr (0.81 g, 0.86 mL, 3.0 equiv); Ti(O*i*Pr)_4_ (0.51 g, 0.53 mL, 1.0 equiv); ArMgBr: 4‐fluoro‐3‐methylphenylmagnesium bromide (1.0 m THF solution, 9.0 mL, 5.0 equiv). ^1^H NMR (399.89 MHz; C_6_D_6_; 295.3 K): *δ* [ppm]=6.97–6.56 (m, 21 H), 1.98 (br, 6 H), 1.96 (br, 6 H), 1.86 (br, 6 H). ^13^C{^1^H} NMR (100.55 MHz; C_6_D_6_; 296.1 K): *δ* [ppm]=161.1, 160.8 (d, ^1^ *J*_CF_=246.0 Hz), 160.7 (d, ^1^ *J*_CF_=245.7 Hz), 160.6 (d, ^1^ *J*_CF_=245.8 Hz), 140.9, 140.3, 139.5 (d, ^4^ *J*_CF_=3.7 Hz), 138.7 (d, ^4^ *J*_CF_=3.8 Hz), 138.3 (d, ^4^ *J*_CF_=3.8 Hz), 135.5, 134.44 (d, ^3^ *J*_CF_=5.0 Hz), 134.41 (d, ^3^ *J*_CF_=5.0 Hz), 134.2 (d, ^3^ *J*_CF_=5.0 Hz), 130.61 (d, ^3^ *J*_CF_=7.8 Hz), 130.58 (d, ^3^ *J*_CF_=7.7 Hz), 130.20 (d, ^3^ *J*_CF_=7.7 Hz), 124.7 (d, ^2^ *J*_CF_=17.4 Hz), 124.5 (d, ^2^ *J*_CF_=17.4 Hz), 124.4 (d, ^2^ *J*_CF_=17.4 Hz), 123.8, 114.84 (d, ^2^ *J*_CF_=22.4 Hz), 114.77 (d, ^2^ *J*_CF_=22.4 Hz), 114.5 (d, ^2^ *J*_CF_=22.3 Hz), 14.5–14.2 (m). ^19^F{^1^H} NMR (376.27 MHz, C_6_D_6_, 295.7 K): *δ* [ppm]=−118.6, −118.8, −118.9. MS (HR‐DART(+)): calcd 780.3065 (C_51_H_40_F_6_N, [*M*+H]^+^), found 780.3053.

**Compound 4**: Colourless solid, 3.8 g, 5.5 mmol, Yield: 55 %. Alkyne: 2,6‐bis((3‐fluorophenyl)ethynyl)pyridine (3.3 g, 10 mmol); *i*PrSS*i*Pr (4.5 g, 4.8 mL, 3.0 equiv); Ti(O*i*Pr)_4_ (2.8 g, 2.9 mL, 1.0 equiv); ArMgBr: 3‐fluorophenylmagnesium bromide (1.0 m THF solution, 50 mL, 5.0 equiv). ^1^H NMR (399.89 MHz; C_6_D_6_; 295.1 K): *δ* [ppm]=6.95–6.55 (m, 25 H), 6.55–6.38 (m, 2 H). ^13^C{^1^H} NMR (100.55 MHz; C_6_D_6_; 295.1 K): *δ* [ppm]=162.97 (d, ^1^ *J*_CF_=245.2 Hz), 162.96 (d, ^1^ *J*_CF_=246.7 Hz), 162.8 (d, ^1^ *J*_CF_=245.9 Hz), 161.1, 145.1 (d, ^3^ *J*_CF_=7.6 Hz), 144.3 (d, ^3^ *J*_CF_=7.6 Hz), 143.9 (d, ^3^ *J*_CF_=7.6 Hz), 141.3 (br), 141.2 (br), 136.0 (br), 129.9 (d, ^3^ *J*_CF_=8.2 Hz), 129.7 (d, ^3^ *J*_CF_=8.2 Hz), 129.6 (d, ^3^ *J*_CF_=8.2 Hz), 127.0 (d, ^4^ *J*_CF_=2.7 Hz), 126.92 (d, ^4^ *J*_CF_=2.7 Hz), 126.85 (d, ^4^ *J*_CF_=2.7 Hz), 124.1, 118.1 (d, ^2^ *J*_CF_=21.8 Hz), 118.0 (d, ^2^ *J*_CF_=21.8 Hz), 117.7 (d, ^2^ *J*_CF_=21.8 Hz), 114.7 (d, ^2^ *J*_CF_=21.4 Hz), 114.4 (d, ^2^ *J*_CF_=21.4 Hz), 114.4 (d, ^2^ *J*_CF_=21.1 Hz). ^19^F{^1^H} NMR (376.27 MHz, C_6_D_6_, 295.1 K): *δ* [ppm]=−112.7 (s, 2 F), −113.10 (s, 1 F), −113.11 (s, 1 F), −113.27 (s, 1 F), −113.28 (s, 1 F). MS (HR‐DART(+)): calcd 696.2126 (C_45_H_28_F_6_N, [*M*+H]^+^), found 696.2116.

**Compound 5**: Colourless solid, 1.7 g, 2.1 mmol, Yield: 46 %. Alkyne: 2,6‐bis((3,5‐difluorophenyl)ethynyl)pyridine (1.6 g, 4.6 mmol); *i*PrSS*i*Pr (2.1 mg, 2.2 mL, 3.0 equiv); Ti(O*i*Pr)_4_ (1.3 mg, 1.4 mL, 1.0 equiv); ArMgBr: 3,5‐difluorophenylmagnesium bromide (1.0 m THF solution, 23 mL, 5.0 equiv). ^1^H NMR (399.89 MHz; C_6_D_6_; 295.3 K): *δ* [ppm]=6.69–6.61 (m, 1 H), 6.49–5.83 (m, 20 H). ^13^C{^1^H} NMR (100.55 MHz; C_6_D_6_; 295.2 K): *δ* [ppm]=164.5–161.6 (m), 159.6, 144.8–144.7 (m), 143.9–143.7 (m), 141.2–141.1 (m), 140.3 (br), 136.5, 124.2, 113.9–113.3 (m), 104.2–103.2 (m). ^19^F{^1^H} NMR (C_6_D_6_, 376.27 MHz, 295.3 K): *δ* [ppm]=−108.6, −109.0 (br), −109.3 (br). MS (HR‐DART(+)): calcd 804.1561 (C_45_H_22_F_12_N, [*M*+H]^+^), found 804.1536.

**Compound 6**: Colourless solid, 0.60 g, 0.65 mmol, Yield: 25 %. Alkyne: 2,6‐bis((4‐(*tert*‐butyl)phenyl)ethynyl)pyridine (1.0 g, 2.6 mmol); *i*PrSS*i*Pr (1.2 g, 1.3 mL, 3.0 equiv); Ti(O*i*Pr)_4_ (0.74, 0.77 mL, 1.0 equiv); ArMgBr: 4‐*tert*‐butylphenylmagnesium bromide (1.0 m THF solution, 13 mL, 5.0 equiv). ^1^H NMR (399.89 MHz; C_6_D_6_; 295.1 K): *δ* [ppm]=7.19–7.16 (m, 3 H), 7.16–7.13 (m, 3 H), 7.11–7.05 (m, 14 H), 7.00–6.94 (m, 4 H), 6.78–6.68 (m, 2 H), 6.68–6.62 (m, 1 H), 1.19 (s, 18 H), 1.09 (s, 18 H), 1.04 (s, 18 H). ^13^C{^1^H} NMR (100.55 MHz; C_6_D_6_; 295.1 K): *δ* [ppm]=162.8, 149.4, 149.2, 149.0, 141.9, 141.8, 141.2, 140.7, 140.3, 135.4, 131.8, 131.6, 131.4, 125.0, 124.8, 124.7, 123.8, 34.6, 34.45, 34.43, 31.6, 31.40, 31.36. MS (HR‐DART(+)):calcd 924.6447 (C_69_H_82_N, [*M*+H]^+^), found 924.6426.

## Conflict of interest

The authors declare no conflict of interest.

## Supporting information

As a service to our authors and readers, this journal provides supporting information supplied by the authors. Such materials are peer reviewed and may be re‐organized for online delivery, but are not copy‐edited or typeset. Technical support issues arising from supporting information (other than missing files) should be addressed to the authors.

Supplementary
