# Supplementary material for: Luminogens for Aggregation‐Induced Emission via Titanium‐Mediated Double Nucleophilic Addition to 2,5‐Dialkynylpyridines: Formation and Transformation of the Emitting Aggregates
Source: Chemistry. 2020 Feb 21;26(19):4269–80. doi: 10.1002/chem.201905611 (PMC7187342; doi:10.1002/chem.201905611)
Supplement: Supplementary file 1 — Supplementary [file CHEM-26-4269-s001.pdf]

CHEMISTRY
A European Journal

Supporting Information

Luminogens for Aggregation-Induced Emission via Titanium-Mediated Double Nucleophilic Addition to 2,5-Dialkynylpyridines: Formation and Transformation of the Emitting Aggregates

Francesco Foschi,[a] Kevin Synnatschke,[b] Sebastian Grieger,[b] Wen-Shan Zhang,[c] Hubert Wadepohl,[a] Rasmus R. Schröder,[c] Claudia Backes,*[b] and Lutz H. Gade*[a]

chem_201905611_sm_miscellaneous_information.pdf

Summary

1. Synthetic procedures and characterization of 2,6-bis(arylethynyl)pyridines: General Procedure 2 (**GP-2**).. **3**

2. ^{1}H NMR and $^{13}C\{^{1}H\}$ NMR spectra of 2,6-bis(1,2,2-triarylvinyl)pyridines.................... **7**

3. Additional information on Compound **2**.. **13**

4. Extinction, absorption and fluorescence emission spectra, DLS experiments..**14**

5. X-ray crystal structure determinations...**24**

6. Wavelength-dependent scattering...**26**

7. References (SI).. **27**

1. Synthetic procedures and characterization of 2,6-bis(arylethynyl)pyridines: General Procedure 2 (GP-2)

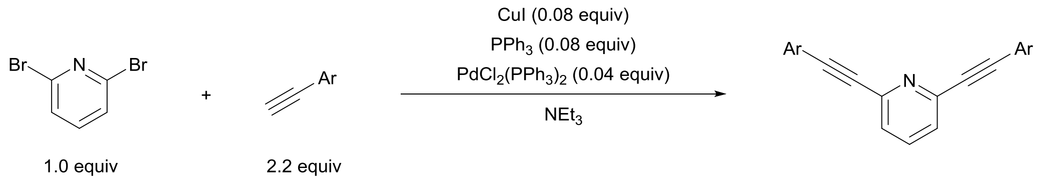

To a stirring mixture of 2,6-dibromopyridine (1.0 equiv), CuI (0.080 equiv), triphenylphosphine (0.080 equiv) and $PdCl_2(PPh_3)_2$ (0.040 equiv) in degassed triethylamine (2 mL each millimole of 2,6-dibromopyridine), an arylacetylene (2.2 equiv) was added dropwise. The mixture was stirred at rt or under reflux for 2–5 days depending on the target compound (see the following analytics), then filtered at rt. The filtrate was adsorbed over celite and purified by column chromatography (SiO_2, eluent gradient: petroleum ether:ethylacetate = 50:1 to 4:1, 0.5 vol% NEt_3), thus yielding the corresponding 2,6-bis(arylethynyl)pyridine as pale yellow solid (28–80%).

2,6-bis((4-fluoro-3-methylphenyl)ethynyl)pyridine, pale yellow solid, 1.2 g, 3.5 mmol, Yield: 56%. 2,6-dibromopyridine (1.5 g, 6.3 mmol); 4-ethynyl-1-fluoro-2-methylbenzene (1.9 g, 1.9 mL); CuI (0.10 g); PPh_3 (0.13 g); $PdCl_2(PPh_3)_2$ (0.18 g); NEt_3 (13 mL). Room temperature, 5 days. ^{1}H NMR (600.13 MHz; C_6D_6; 295.0 K): δ [ppm] = 7.21 – 7.16 (m, 3 H), 7.15 – 7.12 (m, 2 H), 7.08 (d, J= 7.8 Hz, 2 H), 6.81 (t, J= 7.8 Hz, 1 H), 6.64 – 6.54 (m, 2 H), 1.86 (d, J= 1.9 Hz, 6 H). ^{13}C{^{1}H} NMR (150.90 MHz; C_6D_6; 295.0 K): δ [ppm] = 162.0 (d, J= 248.9 Hz), 144.6, 136.1, 135.8 (d, J= 5.6 Hz), 131.7 (d, J= 8.5 Hz), 125.9, 125.7 (d, J= 18.1 Hz), 118.5 (d, J= 3.8 Hz), 115.6 (d, J= 23.3 Hz), 89.0–88.9 (m), 14.1 (d, J= 3.4 Hz). MS (HR-EI(+)): calcd 344.1251 ($C_{23}H_{17}F_2N$, $[M+H]^+$), found 344.1251.

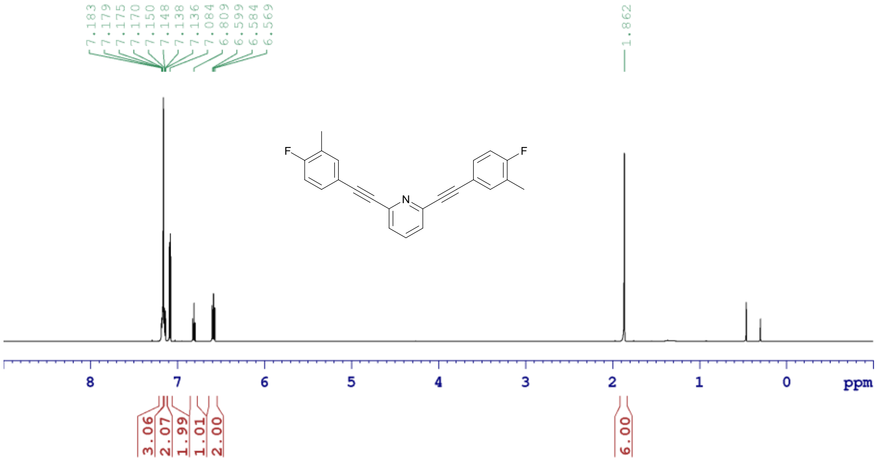

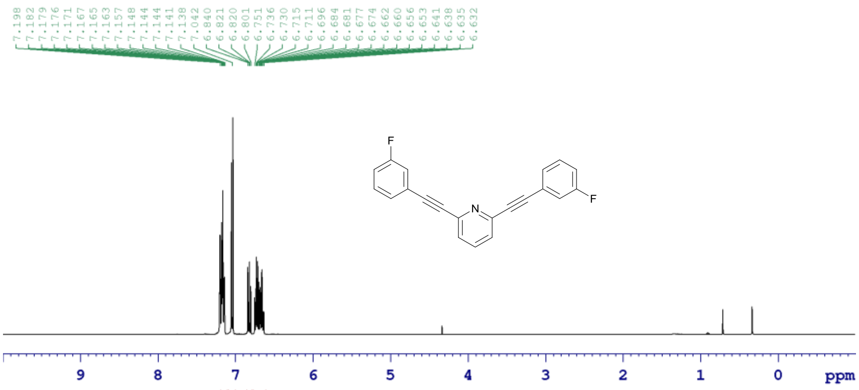

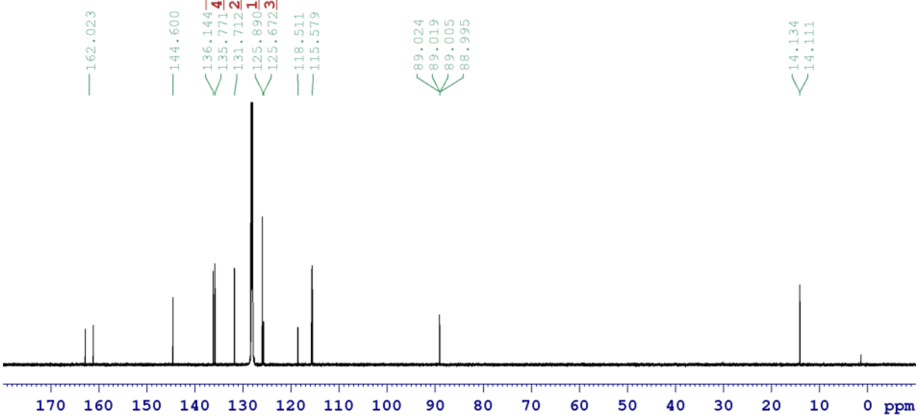

2,6-bis((3-fluorophenyl)ethynyl)pyridine, pale yellow solid, 4.4 g, 14 mmol, Yield: 80%. 2,6-dibromopyridine: (2.6 g, 11 mmol); 1-ethynyl-3-fluorobenzene (2.9 g, 2.8 mL); CuI (0.17 g); PPh_3 (0.23 g); $PdCl_2(PPh_3)_2$ (0.31 g); NEt_3 (22 mL). Room temperature, 4 days. ^{1}H NMR (399.89 MHz; C_6D_6; 295.0 K): δ [ppm] = 7.25 – 7.13 (m, 4 H), 7.04 (d, *J*= 7.8 Hz, 2 H), 6.86 – 6.79 (m, 1 H), 6.77 – 6.61 (m, 4 H). ^{13}C{^{1}H} NMR (100.55 MHz; C_6D_6; 296.4 K): δ [ppm] = 162.7 (d, *J*= 246.7 Hz), 144.1, 136.1, 130.3 (d, *J*= 8.2 Hz), 128.1 (d, *J*= 3.1 Hz), 126.3, 124.5 (d, *J*= 9.5 Hz), 119.0 (d, *J*= 23.0 Hz), 116.5 (d, *J*= 21.1 Hz), 90.1, 88.2 (d, *J*= 3.4 Hz). MS (HR-EI(+)): calcd 316.0938 ($C_{21}H_{13}F_2N$, $[M+H]^+$), found 316.0929.

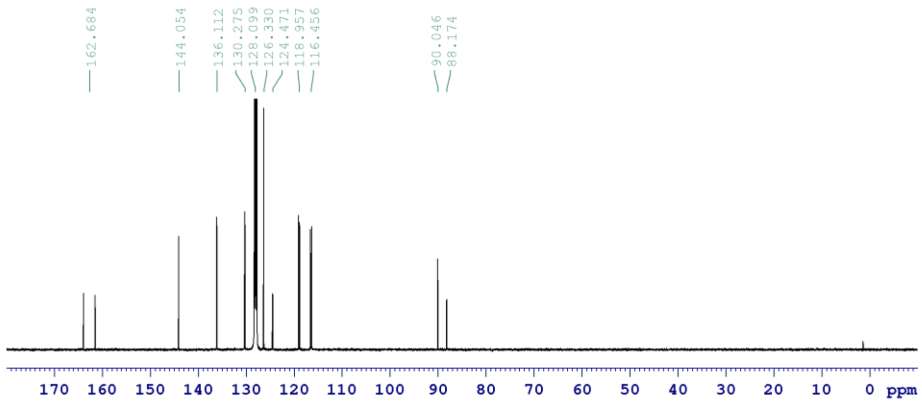

162.684
144.054
136.112
130.275
128.099
126.330
124.471
118.957
116.456
90.046
88.174
170 160 150 140 130 120 110 100 90 80 70 60 50 40 30 20 10 0 ppm

2,6-bis((3,5-difluorophenyl)ethynyl)pyridine, pale yellow solid, 1.3 g, 3.7 mmol, Yield: 60%. 2,6-dibromopyridine: (1.4 g, 6.0 mmol); 1-ethynyl-3,5-difluorobenzene (1.8 g, 1.6 mL); CuI (0.091 g); PPh_3 (0.13 g); $PdCl_2(PPh_3)_2$ (0.17 g); NEt_3 (12 mL). Room temperature, 5 days. ^{1}H NMR (399.89 MHz; C_6D_6; 295.4 K): δ [ppm] = 6.98 (d, *J*= 7.8 Hz, 2 H), 6.91 – 6.64 (m, 5 H), 6.49 – 6.12 (m, 2 H). ^{13}C{^{1}H} NMR (100.55 MHz; C_6D_6; 296.2 K): δ [ppm] = 163.1 (d, *J*= 249.2 Hz), 162.9 (d, *J*= 249.5 Hz), 143.8, 136.3, 126.7, 125.3 – 125.0 (m), 115.2 (d, *J*= 19.3 Hz), 115.1 (d, *J*= 19.3 Hz), 105.6 – 105.1 (m), 90.7, 87.2 – 87.1 (m). MS (HR-EI(+)): calcd 352.0749 ($C_{21}H_{11}F_4N$, $[M+H]^+$), found 352.0745.

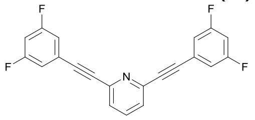

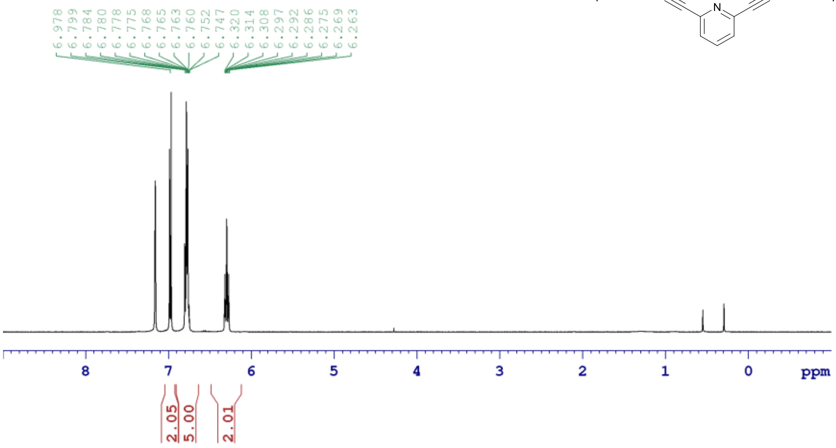

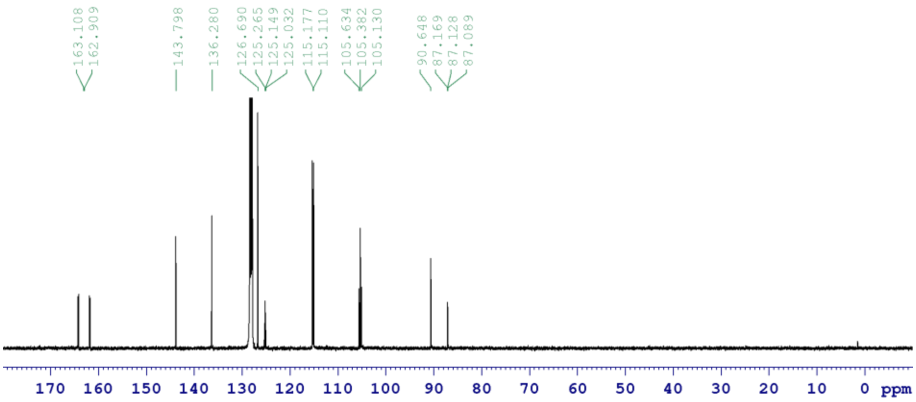

2,6-bis((4-(*tert*-butyl)phenyl)ethynyl)pyridine, pale yellow solid, 1.2 g, 3.0 mmol, Yield: 28%. 2,6-dibromopyridine: (2.6 g, 11 mmol); 1-(*tert*-butyl)-4-ethynylbenzene (3.8 g, 4.3 mL); CuI (0.17 g); PPh_3 (0.23 g); $PdCl_2(PPh_3)_2$ (0.31 g); NEt_3 (22 mL). Reflux, 2 days. ^{1}H NMR (600.13 MHz; C_6D_6; 295.0 K): δ [ppm] = 7.61 – 7.53 (m, 4 H), 7.13 – 7.17 (m, 4 H), 7.05 (d, *J*= 7.8 Hz, 2 H), 6.74 (t, *J*= 7.8 Hz, 1 H), 1.09 (s, 18 H). ^{13}C{^{1}H} NMR (150.90 MHz; C_6D_6; 295.0 K): δ [ppm] = 152.3, 144.7, 136.0, 132.3, 125.88, 125.85, 120.0, 89.9, 89.3, 34.7, 31.1. MS (HR-EI(+)): calcd 392.2378 ($C_{29}H_{31}N$, $[M+H]^+$), found 392.2371.

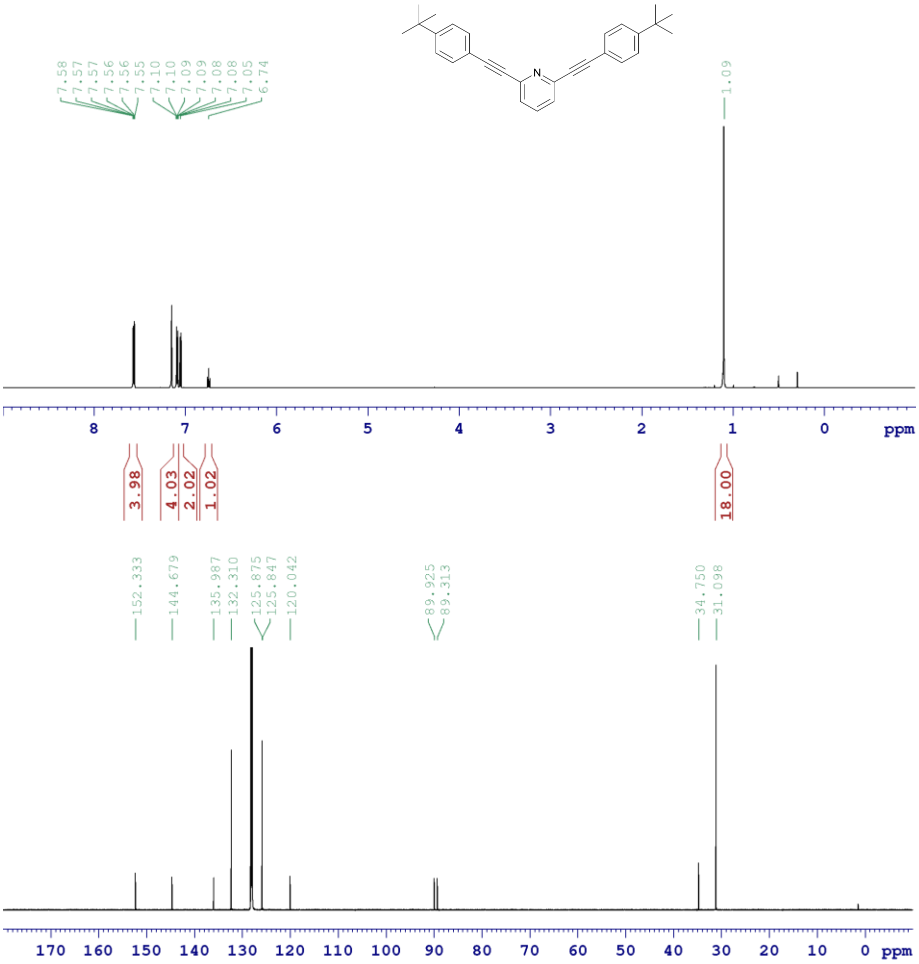

2. ^{1}H NMR and $^{13}C\{^{1}H\}$ NMR Spectra of 2,6-bis(1,2,2-triarylvinyl)pyridines

Compound 1

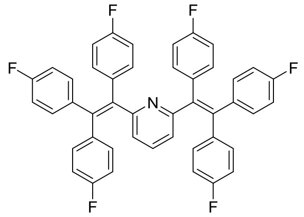

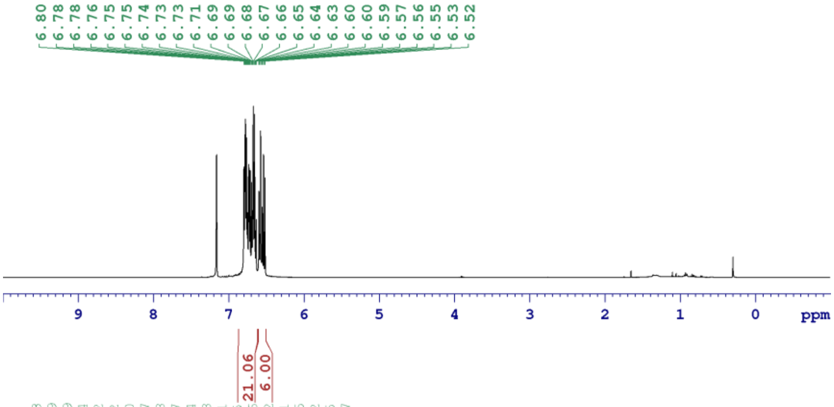

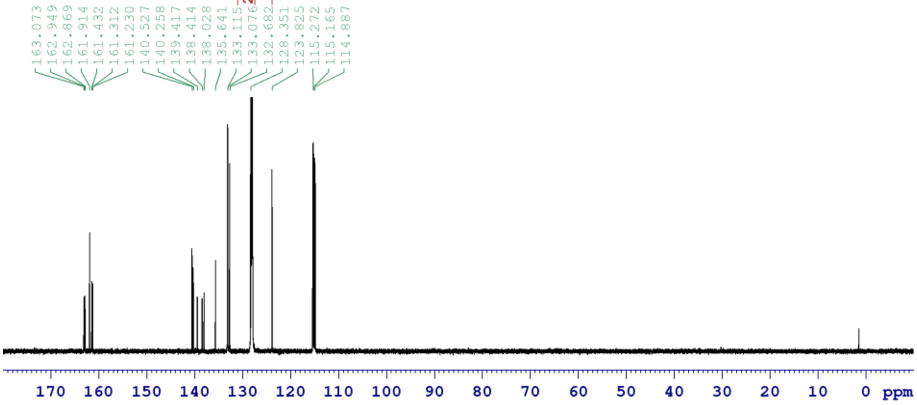

Compound 2

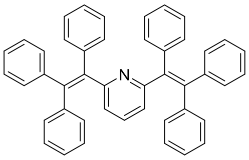
N

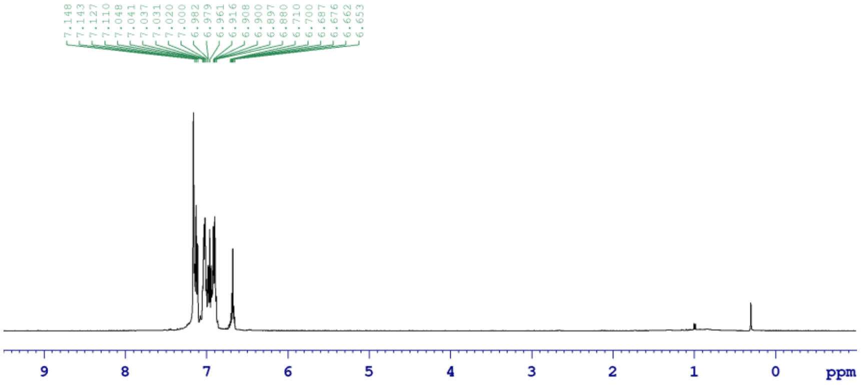
7.148
7.143
7.127
7.110
7.048
7.041
7.037
7.031
7.020
7.000
6.982
6.979
6.961
6.916
6.908
6.900
6.897
6.880
6.710
6.700
6.687
6.676
6.662
6.653
30.45
3.01
9
8
7
6
5
4
3
2
1
0
ppm

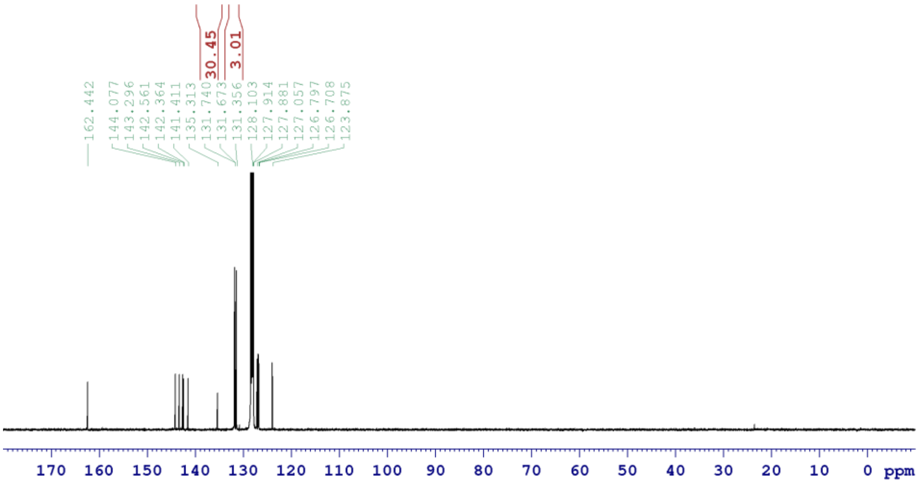
162.442
144.077
143.296
142.561
142.364
141.411
135.313
131.740
131.673
131.356
128.103
127.914
127.881
127.057
126.797
126.708
123.875
170
160
150
140
130
120
110
100
90
80
70
60
50
40
30
20
10
0
ppm

Compound 3

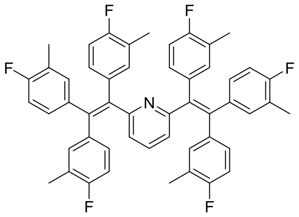

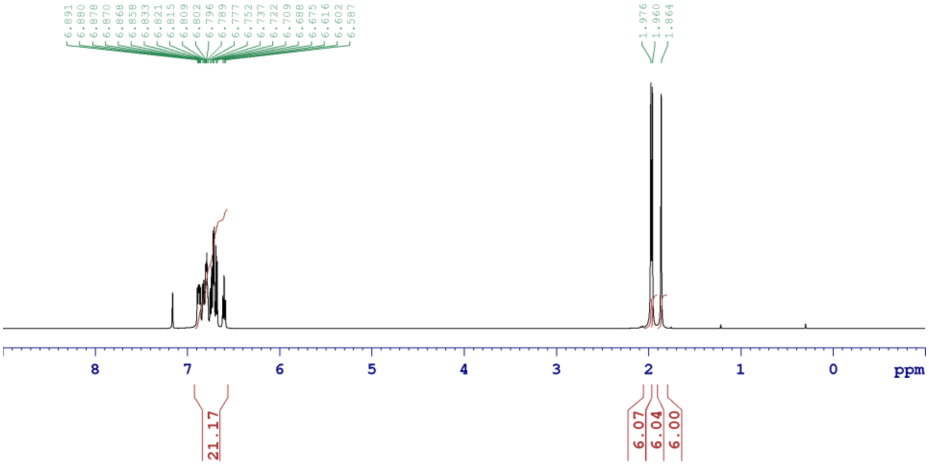

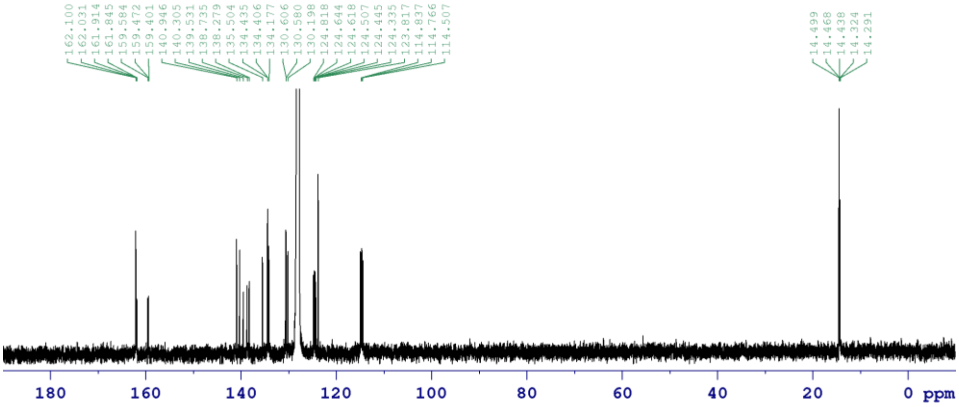

Compound 4

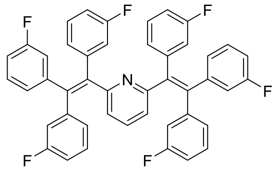
F
F
F
N
F
F
F
F
F

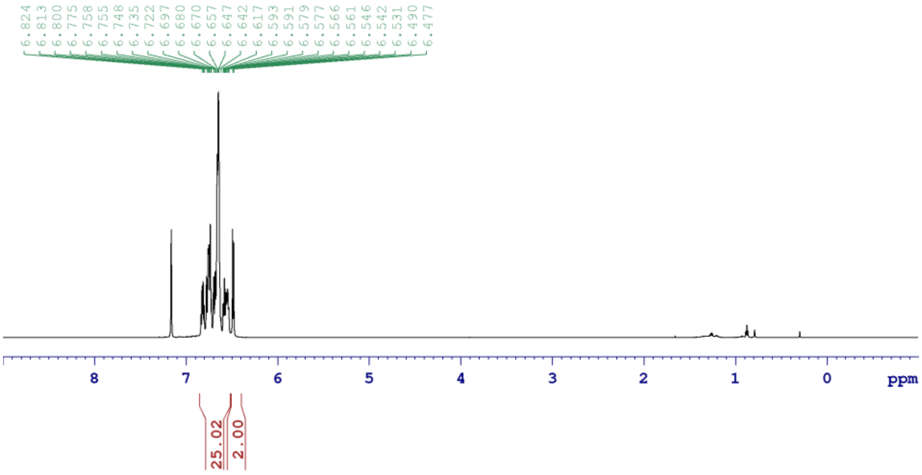
6.824 6.813 6.800 6.775 6.758 6.755 6.748 6.735 6.722 6.697 6.680 6.670 6.657 6.647 6.642 6.617 6.593 6.591 6.579 6.577 6.566 6.561 6.546 6.542 6.531 6.490 6.477
8 7 6 5 4 3 2 1 0 ppm
25.02
2.00

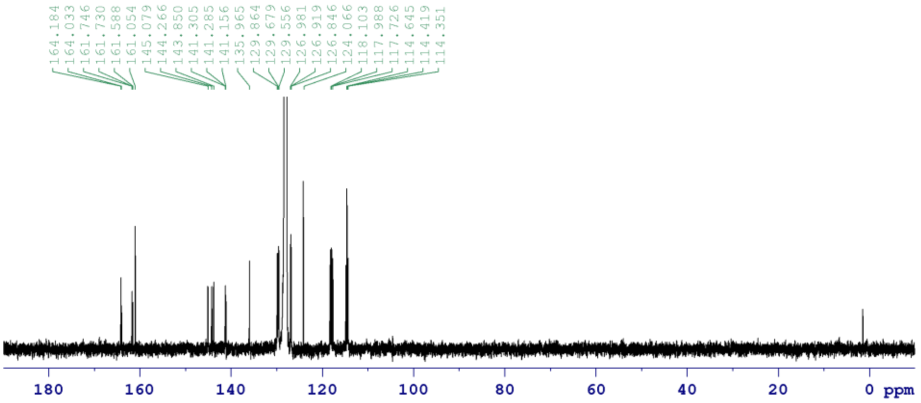
164.184 164.033 161.746 161.730 161.588 161.054 145.079 144.266 143.850 141.305 141.285 141.156 135.965 129.864 129.679 129.556 126.981 126.919 126.846 124.066 118.103 117.988 117.726 114.645 114.419 114.351
180 160 140 120 100 80 60 40 20 0 ppm

Compound 5

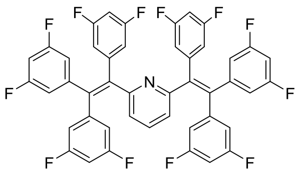
F
F
F
F
F
F
N
F
F
F
F
F
F

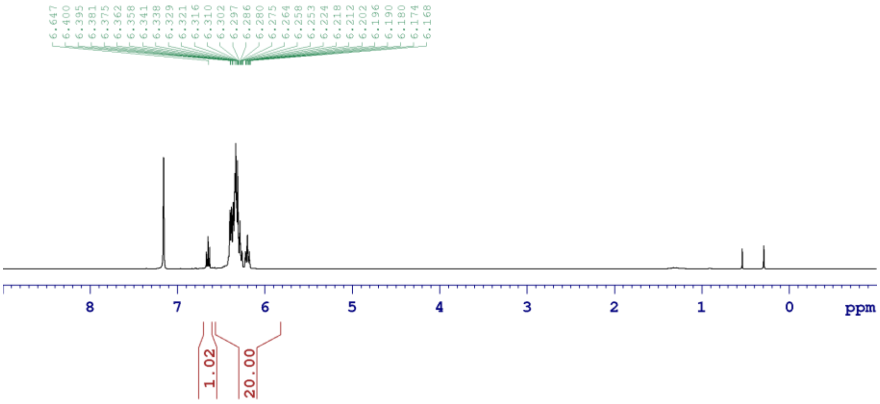
6.647
6.400
6.395
6.381
6.375
6.362
6.358
6.341
6.338
6.329
6.321
6.316
6.310
6.302
6.297
6.286
6.280
6.275
6.264
6.258
6.253
6.224
6.218
6.212
6.202
6.196
6.190
6.180
6.174
6.168
8
7
6
5
4
3
2
1
0
ppm
1.02
20.00

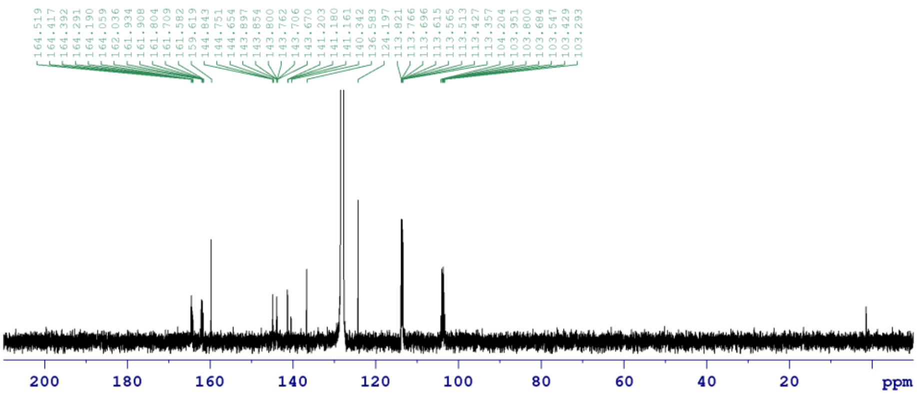
164.519
164.417
164.392
164.291
164.190
164.059
162.036
161.934
161.908
161.804
161.709
161.582
159.619
144.843
144.751
144.654
143.897
143.854
143.800
143.762
143.706
143.670
141.203
141.180
141.161
140.342
136.583
124.197
113.821
113.766
113.696
113.615
113.565
113.513
113.427
113.357
104.204
103.951
103.800
103.684
103.547
103.429
103.293
200
180
160
140
120
100
80
60
40
20
ppm

Compound 6

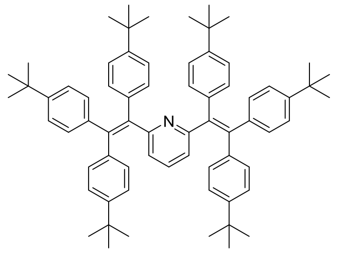
N

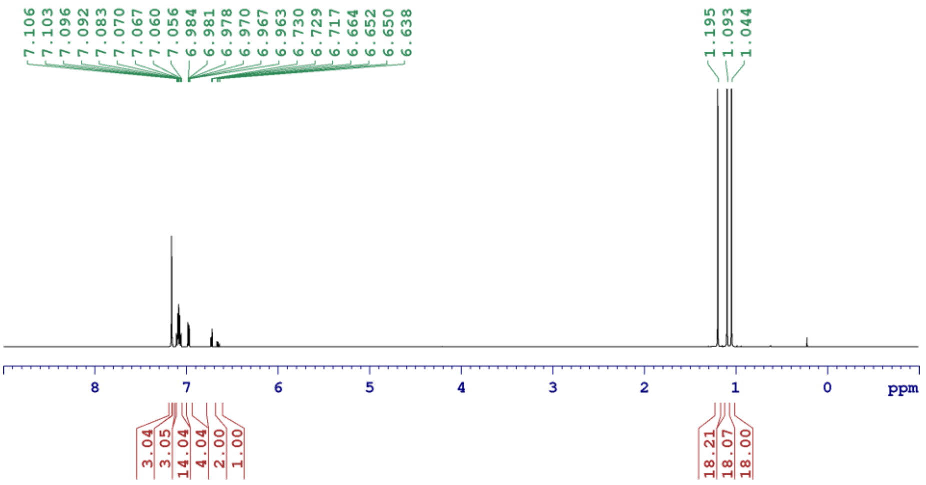
7.106
7.103
7.096
7.092
7.083
7.070
7.067
7.060
7.056
6.984
6.981
6.978
6.970
6.967
6.963
6.730
6.729
6.717
6.664
6.652
6.650
6.638
1.195
1.093
1.044
8
7
6
5
4
3
2
1
0
ppm
3.04
3.05
14.04
4.04
2.00
1.00
18.21
18.07
18.00

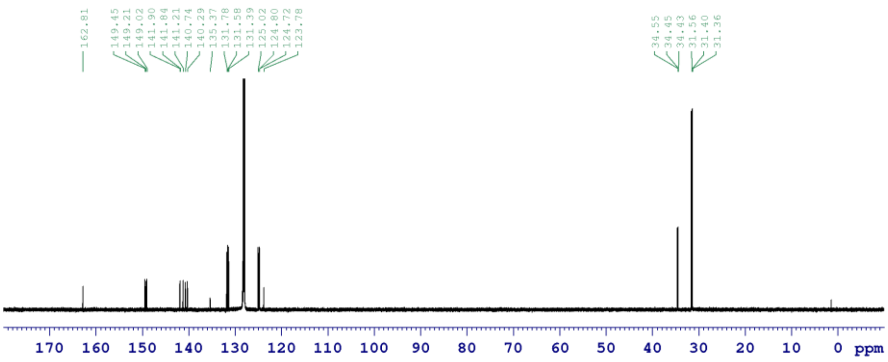
162.81
149.45
149.21
149.02
141.90
141.84
141.21
140.74
140.29
135.37
131.78
131.58
131.39
125.02
124.80
124.72
123.78
34.55
34.45
34.43
31.56
31.40
31.36
170
160
150
140
130
120
110
100
90
80
70
60
50
40
30
20
10
0
ppm

3. Additional information on Compound 2.

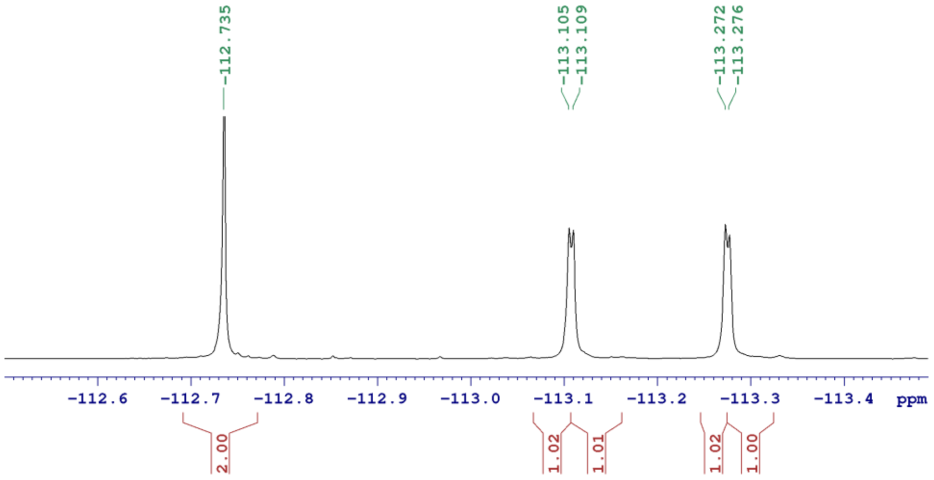

Figure S1. $^{19}F\{^{1}H\}$ NMR spectrum of **4** in C_6D_6. Splitting of singlets suggests the presence of two rotamers of **4** in solution.

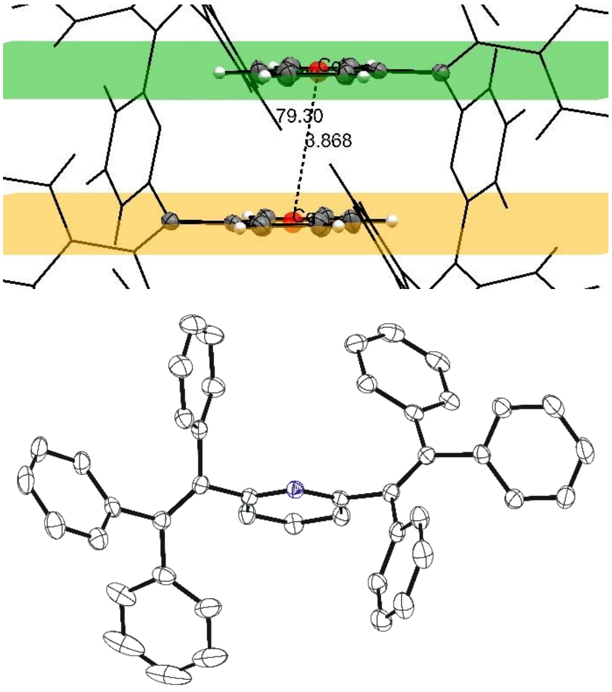

Figure S2. Top: π-π stacking in the X-ray diffraction structure of **2**. Torsion: 79.30°, Cg-Cg' distance: 3.868 Å. Bottom: X-ray diffraction molecular structure of **2**.

4. Extinction, absorption and fluorescence emission spectra, DLS experiments

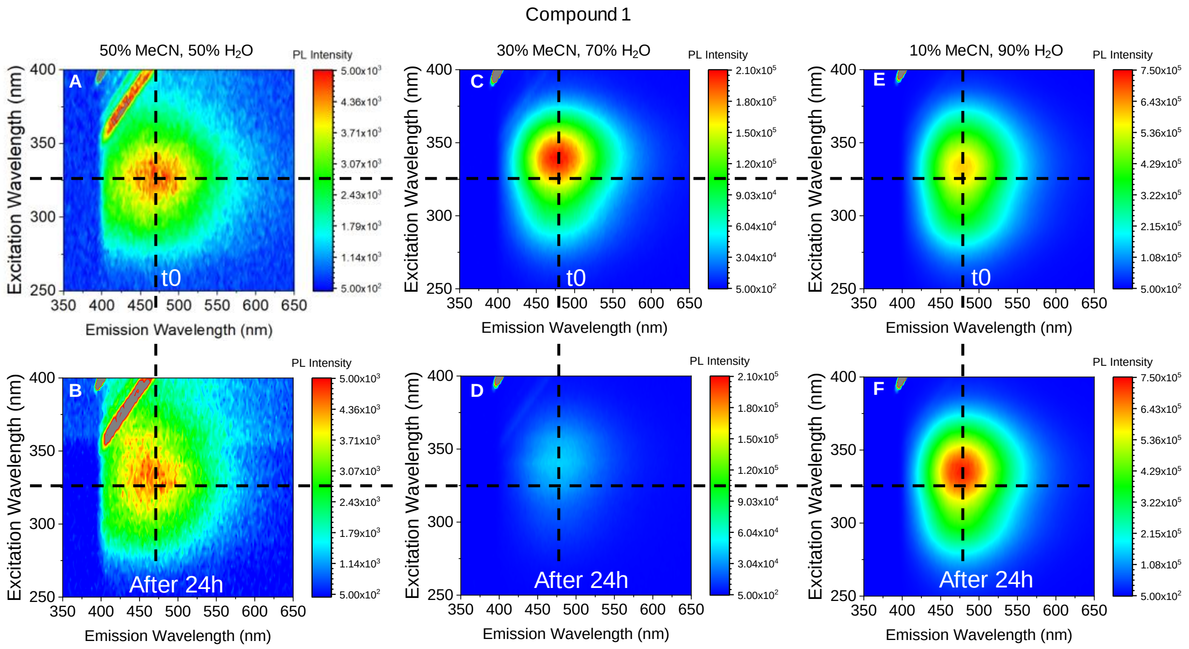

Supporting data for compound 1

Figure S3. Photoluminescence excitation/emission (PLE) maps of **1** for different water/acetonitrile mixtures. A) **1** in 50% v/v H_2O measured right after mixing and B) after 24h. C) **1** in 70% v/v H_2O measured right after mixing and D) after 24h. E) **1** in 90% v/v H_2O measured right after mixing and F) after 24h. The dispersions were kept at rest in quartz cuvettes under light exclusion until the measurements were completed.

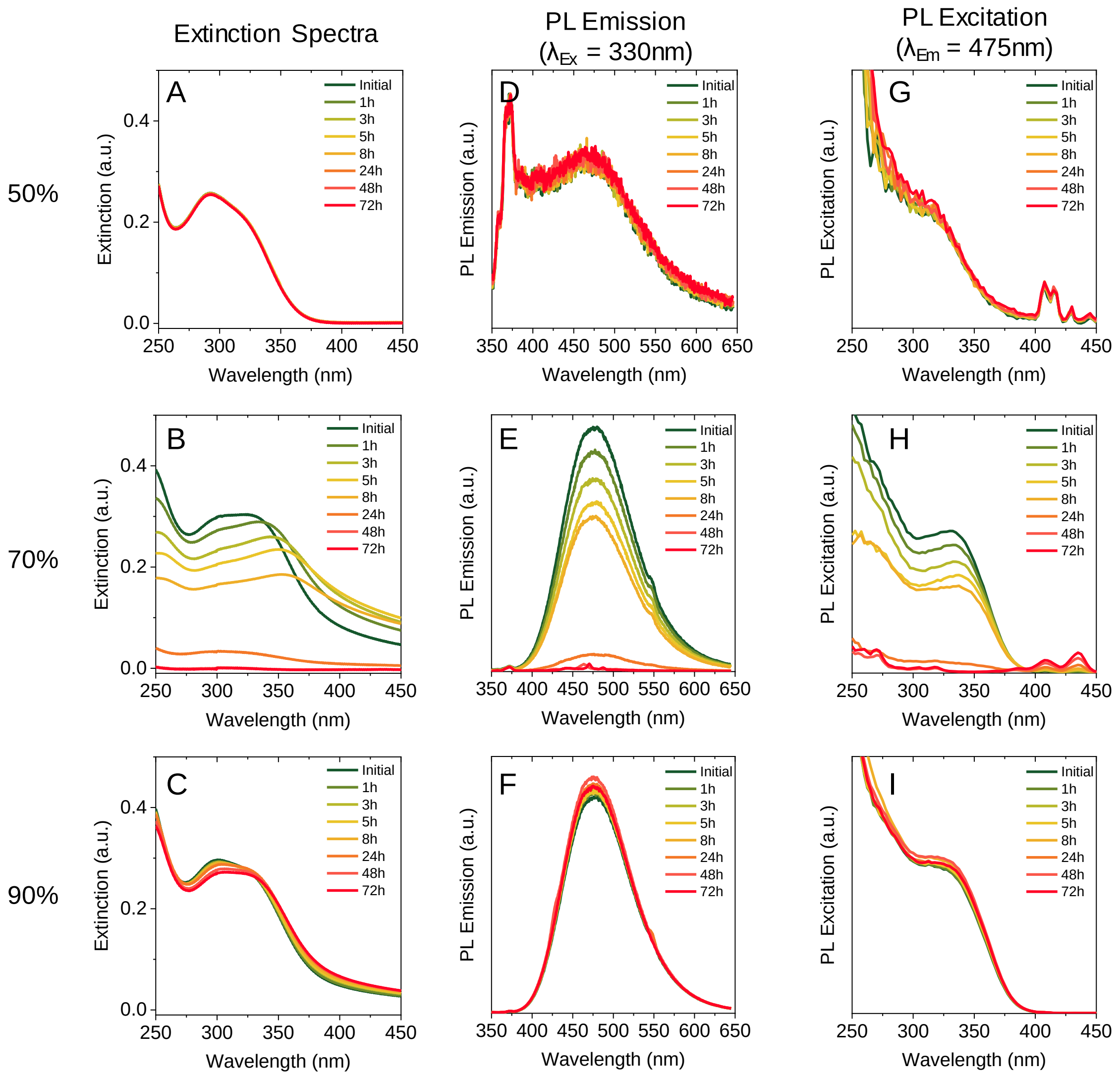

Figure S4. Evolution of extinction, PL emission and excitation spectra of **1** over time for different water/acetonitrile mixtures. A-C) Extinction spectra of **1** in 50, 70 and 90% v/v H_2O, respectively, measured over time. D-F) PL emission of **1** in 50, 70 and 90% v/v H_2O, respectively, measured over time (λ_{Ex} = 330 nm). G-I) PL excitation spectra of **1** in 50, 70 and 90% v/v H_2O, respectively, measured over time (λ_{Em} = 475 nm). No significant changes of the spectra for the 50% v/v H_2O mixture are observed over time, which is consistent with negligible aggregation. For the 70% v/v H_2O mixture, all the spectra change drastically due to aggregation. After 72h most of the material has sedimented, as suggested by a dramatic drop in the extinction and PL intensity. The 90% v/v H_2O mixture shows minor changes over time. The extinction spectra show a slight scattering background. PL emission and excitation show minor changes over time after an initial increase during the first 24h. The dispersions were kept at rest inside quartz cuvettes under light exclusion until the measurements were completed.

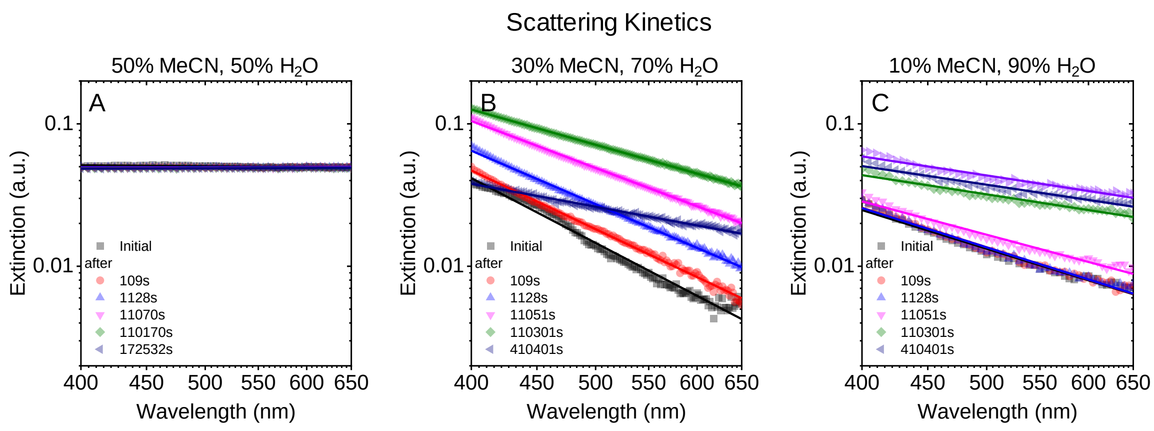

Figure S5. Scattering background in the non-resonant regime of the extinction spectra of **1** over time for different water/acetonitrile mixtures. The linear trend in the non-resonant regime on a double logarithmic scale allows to extract the scattering exponent "-m" from the slope of a line fit (see supplementary section 6 for further explanation). A)-C) Non-resonant scattering contribution and linear fits of 50% (A), 70% (B) and 90% (C) v/v H_2O, respectively. The dispersions were kept at rest in quartz cuvettes under light exclusion until the measurements were completed.

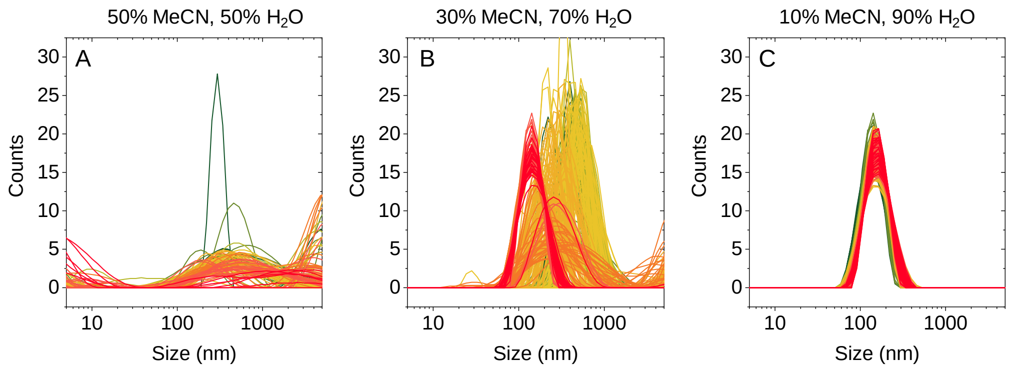

Figure S6. Dynamic light scattering (DLS) experiments of **1** over time. The evolution of the spectra over time is shown for the 50% (A), 70% (B) and 90% (C) v/v H_2O mixtures. Color key for the time evolution: green (t_0) to red (t=4 days). Data are not significant for the 50% v/v H_2O mixture due to insufficient signal-to-noise ratio (A). In the 70% v/v H_2O mixture particles grow over the time approaching an average particle size as big as 530 nm; precipitation then occurs, leaving in suspension only a minor fraction of smaller particles (160 nm ca., red curves). In the 90% v/v H_2O mixture the average particle size grows moderately from 130 nm to 160 nm over time resulting in an overall stable colloid. The dispersions were kept at rest inside quartz cuvettes under light exclusion until the measurements were completed.

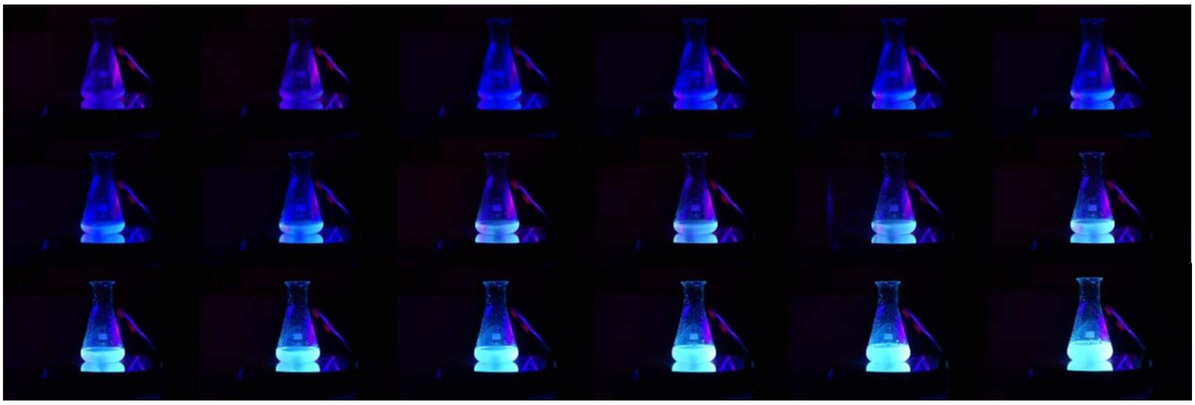

Figure S7. Photographic representation of the viscochromic response of **1** (150μM) in DMSO/glycerol mixtures upon increasing the fraction of glycerol under UV illumination (λ_{exc} = 360 nm). The viscosity of the mixture, and therefore the PL intensity, increase with increasing amount of glycerol added due to aggregation-independent RIM. Top left: DMSO solution of **1**; Bottom right: **1** after addition of glycerol to the final ratio DMSO/glycerol = 1:8 mixture.

Spectral data compounds 1-5

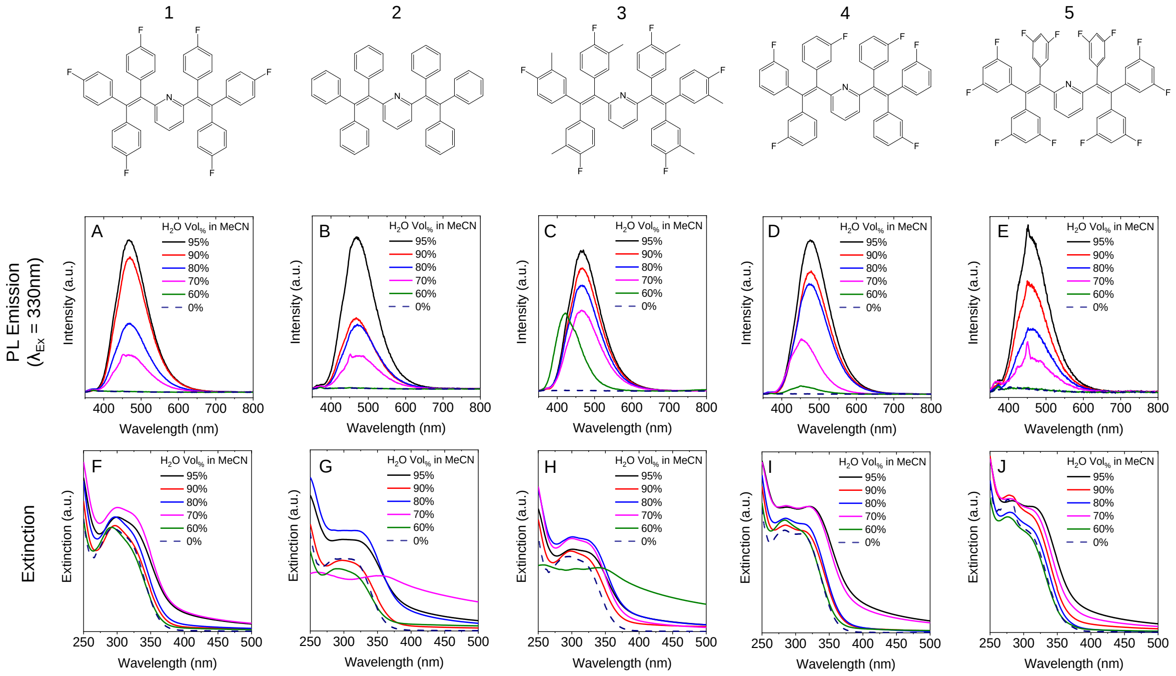

Figure S8. Five different luminogens under investigation. A-E) Photoluminescence (PL) spectra of **1–5** (from left to right) in different water/acetonitrile mixtures. F-J) Extinction spectra of **1–5** in the same solvent mixtures described for the PL spectra.

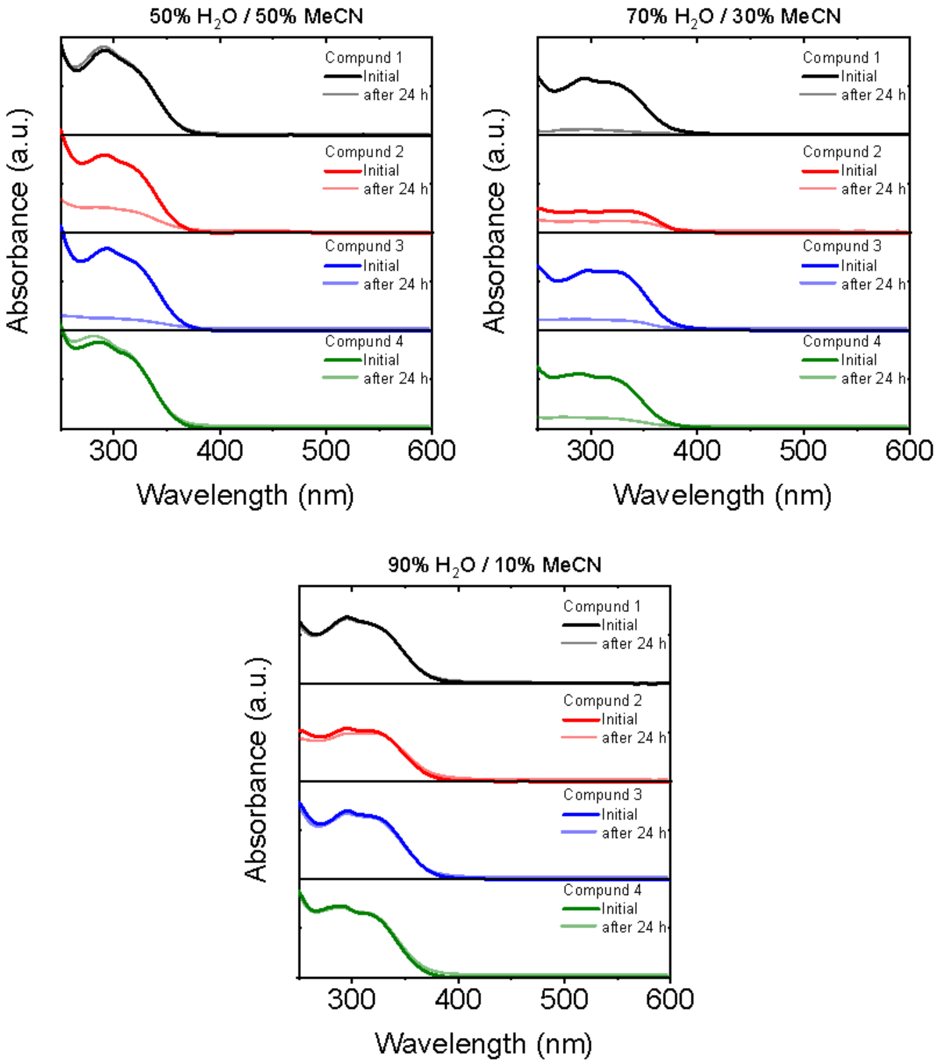

Figure S9. Absorbance spectra of **1–4**. True absorbance spectra, which were measured with the aid of an integrating sphere, show negligible optical density for **1–4** in the non-resonant regime (λ>400nm) for all the solvent mixtures under investigation. As discussed in the main text, the scattering background can be determined as the difference between the extinction spectra (Figures S4, S10–14) and the absorbance spectra shown in this Figure.

PL and Extinction Evolution of 1

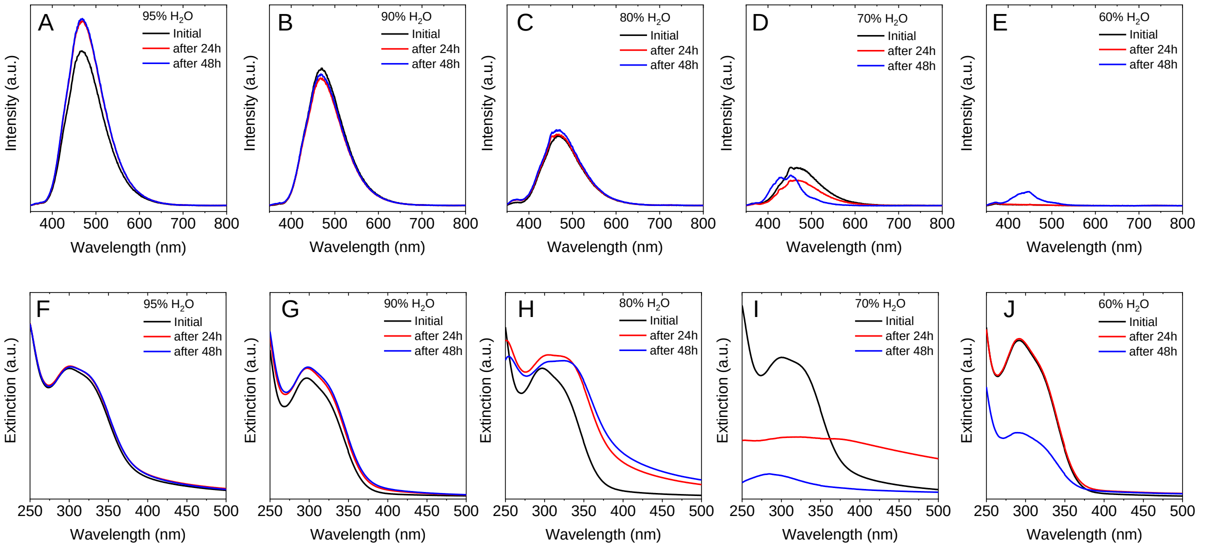

Figure S10. Behaviour of Compound 1 over time. A-E) Photoluminescence spectra and F-J) extinction spectra of **1** for different acetonitrile/water mixtures, measured right after mixing and then after 24 h and 48 h. (A, F) and 90% (B, G) v/v H_2O are relatively stable over 48h, in terms of both emission and extinction, with exception of a moderate increase of the emission intensity over time for the 95% v/v H_2O mixture. The 80% v/v H_2O mixture shows minor changes in the emission spectra (C) but a substantial increase of scattering intensity, along with a pronounced red shift, in the extinction spectra (H). The evolution of the extinction profiles is even more pronounced in the 70% v/v H_2O mixture (I), where a strong increase in the scattering intensity can be observed after 24 h but then a dramatic drop in the overall extinction occurs. Moreover, a pronounced blue shift in the emission (D) can be observed over time in this mixture. A faint emission, which drops further in intensity over time (E), is accompanied by a drop of optical extinction over time for the 60% v/v (H_2O) mixture (J). Notably, the scattering background in the non-resonant regime is in this case negligible.

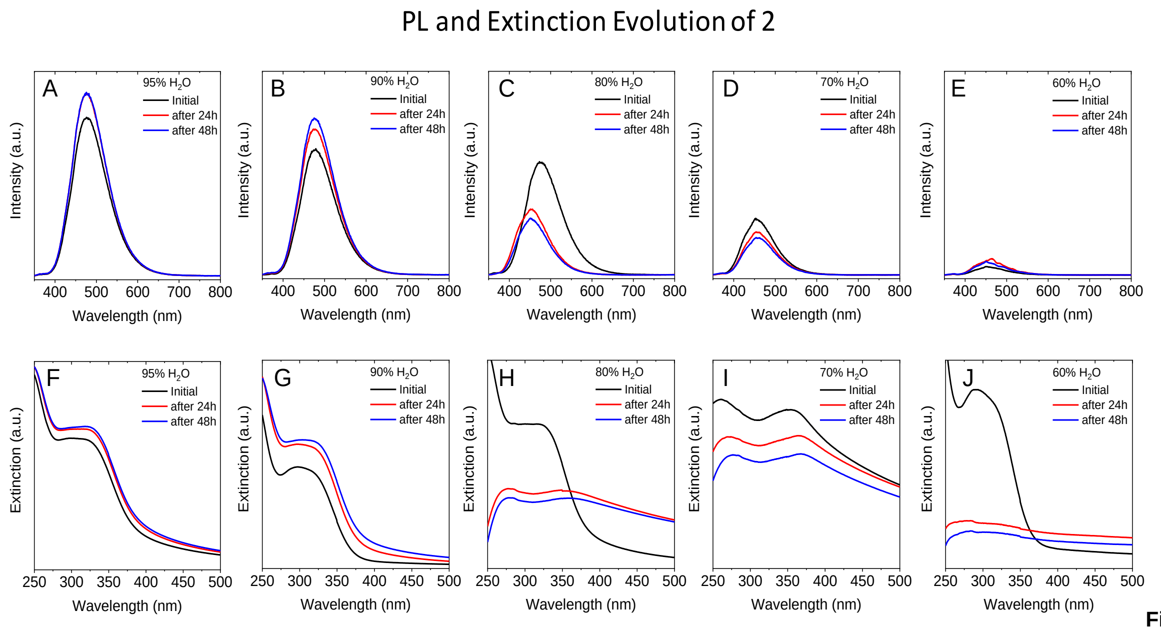

Figure S11. Behaviour of Compound 2 over time A-E) Photoluminescence spectra and F-J) extinction spectra of **2** for different acetonitrile/water mixtures, measured right after mixing and then after 24 h and 48 h. The 95% v/v H_2O mixture (A, F) is the most stable over time. The 90% v/v H_2O mixture (B, G) shows increasing emission intensity and increasing scattering intensity over time. The 80% v/v H_2O mixture (C, H) shows major changes in the emission spectra (C), including a blue shift and a drop in the emission intensity, as well as in the extinction spectra (H), where the scattering contribution rises significantly over time. Similar features can be found in the spectra of **2** in 70% v/v H_2O mixtures (D, I), with exception of the absence of blue shifts in the emission. A faint emission, which drops further in intensity over time (E), is accompanied by a drop of optical extinction over time for the 60% v/v H_2O mixture (J).

PL and Extinction Evolution of 3

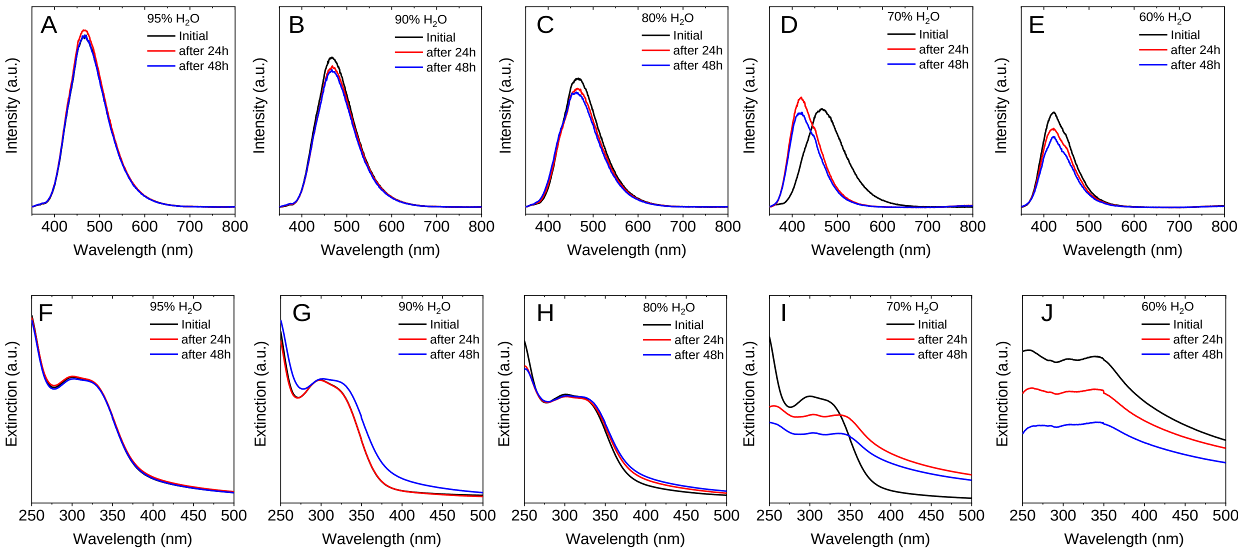

Figure S12. Behaviour of Compound 3 over time. A-E) Photoluminescence spectra and F-J) extinction spectra of **3** for different acetonitrile/water mixtures, measured right after mixing and then after 24 h and 48 h. Mixtures with 95% (A, F) and 90% (B, G) v/v H_2O are relatively stable over 48h, in terms of both emission and extinction, with exception of a moderate increase of the emission intensity over time for the 95% v/v H_2O mixture (A) and of the scattering intensity in the non-resonant regime for the 90% v/v H_2O mixture (G). The 80% v/v H_2O mixture shows minor changes in the emission spectra (C) but a substantial increase of scattering intensity over time in the extinction spectra (H). The 70% v/v H_2O mixture (D, I) shows major changes in the emission spectra (D), involving a strong blue shift, as well as in the extinction spectra (I), where the scattering contribution rises significantly after 24 h. Differently from what observed for the previous compounds, PL intensity of the 60% v/v (H_2O) mixture is not negligible and does not drop significantly over time (E). Notably, the scattering background in the non-resonant regime is remarkable in the freshly prepared 60% v/v H_2O mixture and drops over time (J).

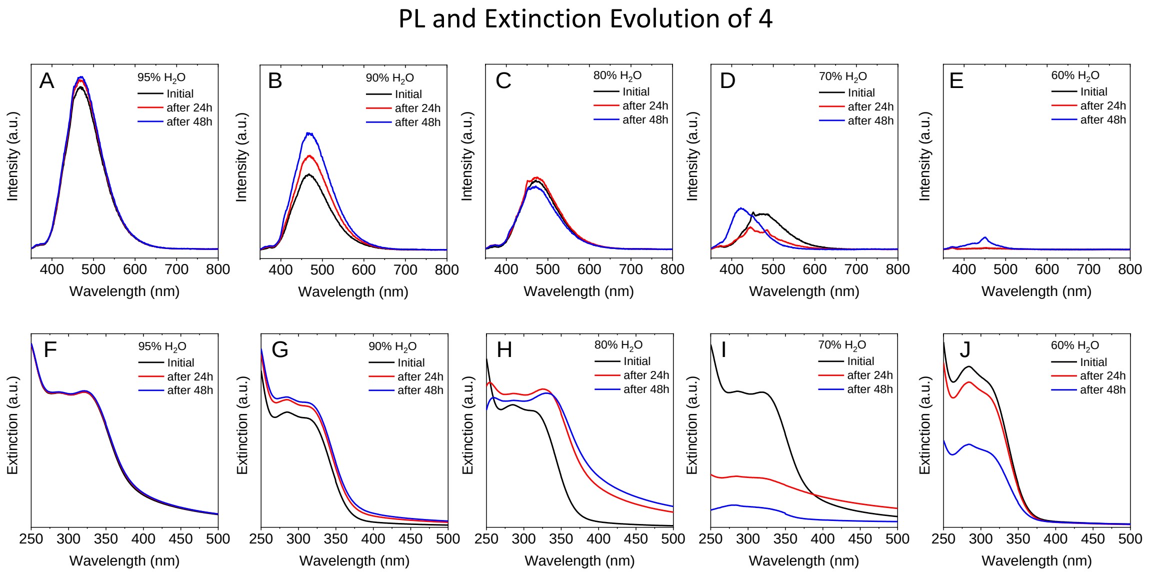

Figure S13. Behaviour of Compound 4 over time. A-E) Photoluminescence spectra and F-J) extinction spectra of **4** for different acetonitrile/water mixtures, measured right after mixing and then after 24 h and 48 h. The 95% v/v H_2O mixture (A, F) is relatively stable over time, while the PL and scattering intensity grow over time for the 90% v/v H_2O mixture (B, G). The 80% v/v H_2O mixture shows minor changes in the emission spectra (C) but a substantial increase of scattering intensity and red shift over time in the extinction spectra (H). The 70% v/v H_2O mixture (D, I) shows major changes in the emission spectra (D), involving a strong blue shift over time, as well as in the extinction spectra (I), where the scattering intensity rises after 24 h and then drops significantly. A drop in the PL (E) and extinction (J) intensities can be observed over time for the 60% v/v H_2O mixture.

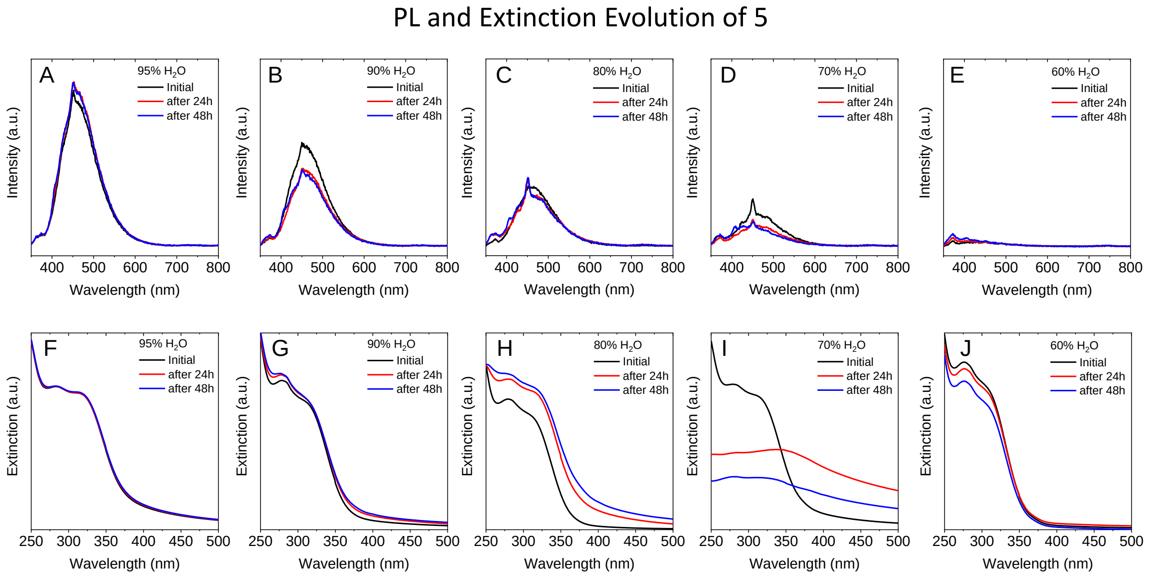

Figure S14. Behaviour of Compound 5 over time. A-E) Photoluminescence spectra and F-J) extinction spectra of **5** for different acetonitrile/water mixtures, measured right after mixing and then after 24 h and 48 h. Mixtures with 95% (A, F) and 90% (B, G) v/v H_2O are relatively stable over 48h, in terms of both emission and extinction, with exception of a moderate increase of scattering intensity over time for the 90% v/v H_2O mixture (G). The 80% v/v H_2O mixture shows minor changes in the emission spectra (C) but a substantial increase of scattering intensity, along with a pronounced red shift, in the extinction spectra (H). The evolution of the extinction profile is even more pronounced in the 70% v/v H_2O mixture (I), where a strong

increase in the scattering intensity can be observed after 24 h but then a dramatic drop in the overall extinction occurs. A faint emission (E) and negligible scattering intensity (J) can be observed for the 60% v/v H_2O mixture.

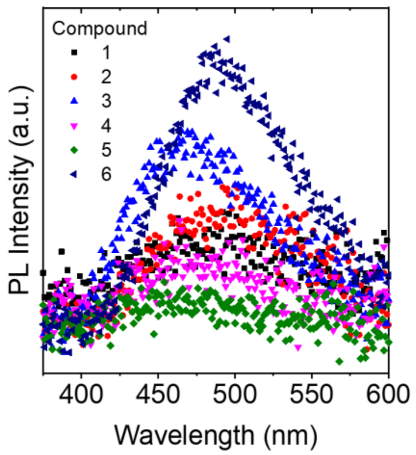

Figure S15. Emission spectra of 1–6 as spin-coated solid films (λ_{exc} = 330 nm).

5. X-ray crystal structure determinations

Crystal data and details of the structure determinations are compiled in Table S1. Full shells of intensity data were collected at low temperature with a Bruker AXS Smart 1000 CCD diffractometer (Mo-$K\alpha$ radiation, sealed X-ray tube, graphite monochromator, compound **4**·C_6D_6) or an Agilent Technologies Supernova-E CCD diffractometer (Mo- or Cu-K_a radiation, microfocus X-ray tube, multilayer mirror optics, all other compounds). Detector frames (typically ω-, occasionally φ-scans, scan width 0.4...1°) were integrated by profile fitting.[S1] Data were corrected for air and detector absorption, Lorentz and polarization effects[S2,S3] and scaled essentially by application of appropriate spherical harmonic functions.[S4-S6] Absorption by the crystal was treated with a semiempirical multiscan method (as part of the scaling process), and augmented by a spherical correction,[S5,S6] or numerically (Gaussian grid).[S6,S7] For datasets collected with the microfocus tubes an illumination correction was performed as part of the numerical absorption correction.[S6]

The structures were solved by ab initio dual space methods (compound **3**·*n*-pentane: SHELXD,[S8] compound **4**·C_6D_6 pentane: VLD procedure[S9]) or by the charge flip procedure[S10] and refined by full-matrix least squares methods based on F^2 against all unique reflections.[S11] All non-hydrogen atoms were given anisotropic displacement parameters. Hydrogen atoms were input at calculated positions and refined with a riding model.[S12] Split atom models were used to refine disordered groups and/or solvent molecules. When found necessary, suitable geometry and adp restraints were applied.[S12,S13] Due to severe disorder and fractional occupancy, electron density attributed to solvent of crystallization was removed from the structures of **3** (*n*-pentane) and **5** (ethyl acetate) with the BYPASS procedure,[S14] as implemented in PLATON (squeeze/hybrid).[S15] Partial structure factors from the solvent masks were included in the refinement as separate contributions to F_{calc}.

To establish comparabiltity with the other structures, the previously published[S16] structure of **1** was re-refined with hydrogen atoms at calculated positions (refined riding).

CCDC 1965537 and 1963833 - 1963835 contains the supplementary crystallographic data for this paper. These data can be obtained free of charge from the Cambridge Crystallographic Data Centre's and FIZ Karlsruhe’s joint Access Service via https://www.ccdc.cam.ac.uk/structures/?.

Table S1. Details of the crystal structure determinations of compounds **1, 3–5**.

	**1**	**3**·*n*-pentane	**4**·C_6D_6	**5**·ethyl acetate
formula	$C_{45}H_{27}F_6N$	$C_{56}H_{51}F_6N$	$C_{51}H_{27}D_6F_6N$	$C_{49}H_{29}F_{12}NO$
crystal system	monoclinic	orthorhombic	orthorhombic	orthorhombic
space group	$P\ 2_1/n$	$P\ 2_12_12_1$	$P\ 2_12_12_1$	*F ddd*
a /Å	16.1155(4)	9.5306(4)	9.452(5) [c]	22.7164(4)
b /Å	10.2929(3)	21.4845(7)	19.596(10) [c]	22.8797(4)
c /Å	20.6155(5)	22.4767(8)	21.407(10) [c]	31.3763(6)
V /$Å^3$	3419.27(14)	4602.3(3)	3965(3) [c]	16307.7(5)
Z	4	4	4	16
M_r	695.67	851.97	779.82	875.73
F_{000}	1432	1792	1600	7136
d_c /Mg·m^{-3}	1.351	1.230	1.306	1.427
μ /mm^{-1}	0.101	0.715	0.094	0.123
max., min. transmission factors	1.000, 0.804 [a]	1.000, 0.806 [b]	0.862, 0.827 [b]	1.000, 0.733 [b]
X-radiation, λ /Å	Mo-*K*α, 0.71073	Cu-*K*α, 1.54184	Mo-*K*α, 0.71073	Mo-*K*α, 0.71073
data collect. temperat. /K	120(1)	120(1)	100(1)	120(1)
θ range /°	2.2 to 30.6	2.8 to 67.3	2.2 to 30.6	2.2 to 32.6
index ranges *h*, *k*, *l*	±23, ±14, ±29	±11, ±25, ±26	±13, ±27, ±30	±34, ±34, ±47
reflections measured	73029	125836	98434	88584
unique [R_{int}]	10459 [0.0795]	8247 [0.2048]	12146 [0.0687]	7458 [0.0838]
observed [$I \geq 2\sigma(I)$]	6833	4834	8267	4780
data / restraints /parameters	10459 / 0 / 469	8247 / 160 / 595	12146 / 19 / 573	7458 / 0 / 263
GooF on F^2	1.030	0.958	1.015	1.048
R indices [$F > 4\sigma(F)$] $R(F)$, $wR(F^2)$	0.0575, 0.1130	0.0550, 0.1147	0.0583, 0.1389	0.0544, 0.1301
R indices (all data) $R(F)$, $wR(F^2)$	0.0969, 0.1293	0.1145, 0.1409	0.1000, 0.1623	0.0909, 0.1458
absolute structure parameter		0.10(11)	-0.3(2)	
largest residual peaks /e·$Å^{-3}$	0.346, -0.245	0.169, -0.180	0.416, -0.350	0.317, -0.224
CCDC deposition number	1965537	1963833	1963834	1963835

[a] Semi-empirical absorption correction; [b] numerical absorption correction; [c] standard uncertainties include systematic error contributions from Monte Carlo simulations.

Deposition numbers for 1: 1817691 (old) and 1965537 (present), 2·*n*-pentane: 1817692.[S16]

6. Wavelength-dependent scattering

While light scattering is typically investigated as function of scattering angle,[S17] its wavelength dependence is much less explored. However, this wavelength dependence is of interest, as it contributes to the extinction spectra and is thus readily accessible experimentally. The wavelength dependent scattering can be described by the *Mie* theory.[S18] The mathematical framework of this theory is rather complex so that various approximations have been derived. Probably the most well-known approximation is the *Rayleigh* approximation [S19], which describes the scattering coefficient as function of wavelength ($\sigma(\lambda)$) according to equation 1:

$$\sigma(\lambda) = \frac{4\pi^4}{\rho D}\left[\frac{(n/n_0)^2-1}{(n/n_0)^2+2}\right]^2\left(\frac{\lambda}{D}\right)^{-4} \quad (1)$$

where D is the particle diameter, ρ is the sphere density and n and n_0 are the refractive indices inside and outside the sphere (the scattering coefficient is found by dividing the Rayleigh scattering cross-section by the sphere mass). Importantly, equation 1 strictly only holds for spheres with diameters $D<\lambda/10$. When considering molecular solutions with D<0.5 nm, the scattering coefficient over the entire spectral region is very small ($<10^{-5}$ $L/g^{-1}m^{-1}$ with ρ=1000 kg/m^3, n=1.5, n_0=1.3) and its contribution to extinction spectra can be neglected. However, this is not the case for colloids.

For larger particles with $D > \lambda/\pi$, the *van Hulst* approximation [S20] applies which can also be expressed in the form of the scattering efficient (derivation see [S21]) according to equation 2:

$$\sigma(\lambda) \approx \frac{4\pi^2(n/n_0-1)^2}{\rho D}\left(\frac{\lambda}{D}\right)^{-2} \quad (2)$$

Both approximations show that the wavelength dependent scattering coefficient can be described as a powerlaw with exponents of -4 in the case of particles with diameters $D<\lambda/10$ and -2 for $D > \lambda/\pi$. Accordingly, taking a wavelength of 800 nm, we would expect a scattering exponent, $-m$, of 4 for spheres < 80 nm and 2 for > 250 nm. Importantly, many colloids, such as these investigated here, have intermediate sizes where neither approximation is applicable. As elaborated recently for platelet-like colloids[S21], this intermediate particle size regime is characterized by exponents $2<-m<4$ with a strong dependence of the exponent on the nanoparticle projected area. As such, the scattering exponent which is accessible from extinction spectra (Figure S5) can be used as a measure for the particle size of the colloids.

References (SI)

S1 K. Kabsch, in: M. G. Rossmann, E. Arnold (eds.), *"International Tables for Crystallography" Vol. F*, Ch. 11.3, Kluwer Academic Publishers, Dordrecht, The Netherlands, **2001**.

S2 *SAINT*, Bruker AXS GmbH, Karlsruhe, Germany **1997-2019**.

S3 *CrysAlisPro*, Agilent Technologies UK Ltd., Oxford, UK **2011**-**2014** and Rigaku Oxford Diffraction, Rigaku Polska Sp.z o.o., Wrocław, Poland **2015**-**2019**.

S4 R. H. Blessing, *Acta Cryst.* **1995**, *A51*, 33–38.

S5 (a) G. M. Sheldrick, *SADABS*, Bruker AXS GmbH, Karlsruhe, Germany **2004-2014**; (b) L. Krause, R. Herbst-Irmer, G. M. Sheldrick, D. Stalke, *J. Appl. Cryst.* **2015**, *48*, 3–10.

S6 *SCALE3 ABSPACK, CrysAlisPro*, Agilent Technologies UK Ltd., Oxford, UK **2011**-**2014** and Rigaku Oxford Diffraction, Rigaku Polska Sp.z o.o., Wrocław, Poland **2015**-**2019**.

S7 W. R. Busing, H. A. Levy, *Acta Cryst.* **1957**, *10*, 180–182.

S8 (a) G. M. Sheldrick, *SHELXD*, University of Göttingen and Bruker AXS GmbH, Karlsruhe, Germany **2000**-**2013**; (b) G. M. Sheldrick, H. A. Hauptman, C. M. Weeks, R. Miller, I. Usón, *Ab initio phasing*, in: M. G. Rossmann, E. Arnold (eds.) *International Tables for Crystallography*, Vol. *F*, pp. 333–351, IUCr and Kluwer Academic Publishers, Dordrecht, The Netherlands, **2001**.

S9 (a) M. C. Burla, R. Caliandro, B. Carrozzini, G. L. Cascarano, C. Cuocci, C. Giacovazzo, M. Mallamo, A. Mazzone, G. Polidori, *SIR2014*, CNR IC, Bari, Italy, **2014**; (b) M. C. Burla, R. Caliandro, B. Carrozzini, G. L. Cascarano, C. Cuocci, C. Giacovazzo, M. Mallamo, A. Mazzone, G. Polidori, *J. Appl. Cryst.* **2015**, *48*, 306–309.

S10 (a) L. Palatinus, *SUPERFLIP*, EPF Lausanne, Switzerland and Fyzikální ústav AV ČR, v. v. i., Prague, Czech Republic, **2007**-**2014**; (b) L. Palatinus, G. Chapuis, *J. Appl. Cryst.* **2007**, 40, 786–790.

S11 (a) G. M. Sheldrick, *SHELXL-20xx*, University of Göttingen and Bruker AXS GmbH, Karlsruhe, Germany **2012**-**2018**; (b) W. Robinson, G. M. Sheldrick in: N. W. Isaaks, M. R. Taylor (eds.) "*Crystallographic Computing 4"*, Ch. 22, IUCr and Oxford University Press, Oxford, UK, **1988**; (c) G. M. Sheldrick, *Acta Cryst.* **2008**, *A64*, 112–122; (d) G. M. Sheldrick, *Acta Cryst.* **2015**, *C71*, 3–8.

S12 (a) J. S. Rollett in: F. R. Ahmed, S. R. Hall, C. P. Huber (eds.) „*Crystallographic Computing"* p. 167, Munksgaard, Copenhagen, Denmark, **1970**; (b) D. Watkin in: N. W. Isaaks, M. R. Taylor (eds.) „*Crystallographic Computing 4"*, Ch. 8, IUCr and Oxford University Press, Oxford, UK, **1988**; (c) P. Müller, R. Herbst-Irmer, A. L. Spek, T. R. Schneider, M. R. Sawaya in: P. Müller (ed.) *"Crystal Structure Refinement"*, Ch. 5, Oxford University Press, Oxford, UK, **2006**; (d) D. Watkin, *J. Appl. Cryst.* **2008**, *41*, 491–522.

S13 A. Thorn, B. Dittrich, G. M. Sheldrick, *Acta Cryst.* **2012**, *A68*, 448–451.

S14 (a) P. v. d. Sluis, A. L. Spek, *Acta Cryst.* **1990**, *A46*, 194–201; (b) A. L. Spek, *Acta Cryst.* **2015**, *C71*, 9–18.

S15 (a) A. L. Spek, *PLATON*, Utrecht University, The Netherlands; (b) A. L. Spek, *J. Appl. Cryst*. **2003**, *36*, 7–13.

S16 F. Foschi, T. Roth, M. Enders, H. Wadepohl, E. Clot, L. H. Gade, *Chem. Commun.* **2018**, *54*, 2228–2231.

S17 H. N. Frock, Particle Size Determination Using Angular Light Scattering. In *Particle Size Distribution*, American Chemical Society: **1987**; Vol. 332, pp 146–160.

S18 G. Mie, *Annalen der Physik* **1908,** 330, 377–445.

S19 L. X. Rayleigh, On the electromagnetic theory of light. *The London, Edinburgh, and Dublin Philosophical Magazine and Journal of Science* **1881,** 12, 81–101.

S20 H. C. Hulst, *Light Scattering by Small Particles*. Dover Publications: New York, **1981**.

S21 A. Harvey, C. Backes, J. B. Boland, X. He, A. Griffin, B. Szydlowska, C. Gabbett, J. F. Donegan, J. N. Coleman, *Nat. Commun.* **2018**, *9*, 4553.
